# Glutaminolysis and mTOR-C/EBPβ Signaling Drives the Differentiation of Novel IL-10-Producing Regulatory T Cells

**DOI:** 10.3390/ijms27198447

**Published:** 2026-09-22

**Authors:** Masaki Tajima, Ivan J. Fuss, Naoko Ikuta, Liming Mao, Timothy G. Myers, Paul J. Gardina, Atsushi Kitani, Warren Strober

**Affiliations:** 1 Mucosal Immunity Section, Laboratory of Clinical Immunology and Microbiology, National Institute of Allergy and Infectious Diseases, National Institutes of Health, Bethesda, MD 20892, USA; ifuss@niaid.nih.gov (I.J.F.); immao@ntu.edu.cn (L.M.); akitani@mail.nih.gov (A.K.); 2 Division of Integrated High-Order Regulatory Systems, Center for Cancer Immunotherapy and Immunobiology, Graduate School of Medicine, Kyoto University, Kyoto 606-8501, Japan; 3 Department of Immunology, Institute of Biomedical Research and Innovation, Foundation for Biomedical Research and Innovation at Kobe, Kobe 650-0047, Japan; n-ikuta@fbri.org; 4 Genomic Technologies Section, Research Technologies Branch, National Institute of Allergy and Infectious Diseases, National Institutes of Health, Bethesda, MD 20892, USA; tgmyers@niaid.nih.gov (T.G.M.); paul.gardina@nih.gov (P.J.G.)

**Keywords:** IL-10, C-type lectins, glutaminolysis, mTOR, ActRIB (ALK4), *C. albicans* infection, asthma

## Abstract

Here we report that Dectin-1-stimulated dendritic cells (DCs) induce unique IL-10-producing T cells with strong regulatory function during fungal infection or experimental asthma. These T cells, which we call Dect-10 T cells, are induced by DC-initiated signaling that sets in motion two complementary mechanisms of IL-10-producing T cell differentiation. The first involves induction of nascent Dect-10 T cell IL-4 synthesis and attendant GATA3 production, the latter being a factor that exerts canonical and epigenetic transcriptional function at separate IL-10 promoter sites. The second involves TGFβ superfamily signaling associated with induction of glutaminolysis and generation of alpha-ketoglutarate, a factor that induces rapamycin-sensitive TORC1 activation; in turn, such activation causes the translation of the LIP isoform of C/EBPβ and the formation of a CREB1-LIP complex that binds to adjacent sites on the IL-10 promoter to facilitate the transactivation of the IL10 gene. Dect-10 T cell regulatory function is manifested through its capacity to down-regulate normally protective immune responses to hyphal *C. albicans* renal infection. In addition, Dect-10 T cells, in accord with their IL-4-dependent genesis, suppress dust mite-induced experimental asthma.

## 1. Introduction

IL-10 is produced by various types of T helper and T regulatory cells, attesting to its role in regulating the function of either the T cell producing it or the function of other cells present in its immediate milieu [1,2]. The magnitude of IL-10 production in these T cell subsets is governed by molecular mechanisms involved in the transcription of the IL-10 gene, including those applicable to most or all T cell subsets or, alternatively, those applicable only to T cells of a specific subset such as the mechanisms guiding IL-10 production only in Th2 cells or Tr1 cells [3]. IL-10 transcription by Th2 cells is driven by IL-4-induced Th2-type transcription factors, STAT6 and GATA3, both acting to cause epigenetic changes in the IL-10 gene locus rather than as transcription factors per se [4,5,6]. In addition, it is driven by c-Maf, a transcription factor that up-regulates both the IL-4 and IL-10 genes and binds to the promoter of both genes [7,8]. Finally, it is driven by IRF4 and Etv5, factors that bind to the IL-10 promoter and/or a conserved IL-10 gene enhancer site [9,10]. In contrast, IL-10 production in Tr1 cells is driven by factors that induce STAT3 expression, notably IL-10 itself, IL-27 or IL-6; this is then followed by the STAT3 induction of c-Maf and AhR, factors that bind to the IL-10 promoter separately or in concert to induce IL-10 transcription [11,12,13,14,15,16]. Thus, while IL-10 production in Th2 cells and Tr1 cells both involve the transcriptional activity of c-Maf, this factor functions in a completely different epigenetic milieu in the two types of cells and in association with unique cofactors.

In the present study we describe a new T cell subset producing high levels of IL-10 that are induced by inhibin beta A-producing Dectin-1-stimulated dendritic cells (DCs) that we have termed Dect-10 T cells. In an extensive analysis, we show that such induction results in a T cell subset that produces IL-10 as a result of a novel transcriptional mechanism that is distinct from that inducing IL-10 in Tr1 cells, Th2 cells or other T cell subsets. It involves first IL-4-mediated GATA3 induction and second TGFβ receptor family signaling, the latter enabling glutaminolysis, leading to mTORC1 activation and the expression of a C/EBPβ isoform/phospho-CREB complex that binds to adjacent sites on the GATA3-modified IL-10 promoter. Notably, the fact that IL-10 transcription in this cell is dependent on glutaminolysis/mTORC1 and independent of c-Maf, a key factor involved in both Th2 and Tr1 IL-10 transcription, as mentioned above, offers strong support for the view that this cell is indeed a new IL-10-producing T cell subset.

The regulatory potential of IL-10-producing T cells originating from Dectin-1 signaling is indicated by the fact that their absence leads to improved survival from potentially lethal renal *C. albicans* infection and increased clearance of organisms from the site of infection, both indicative of a more robust effector cell immune response due to IL-10 down-regulation. In addition, the *in vivo* induction of Dect-10 T cells with Dectin-1 ligand in mice with dust-mite-induced experimental asthma or the administration of Dect-10 T cells to these mice greatly reduces asthmatic inflammation. Thus, Dect-10 T cell regulation is real and has effects on both infection-induced and sterile inflammation, both in the service of limiting potentially destructive inflammation.

## 2. Results

### 2.1. Stimulation of Dendritic Cells with C-Type Lectin Ligands Leads to the Induction of T Cells Producing High Levels of IL-10

In studies designed to explore Dectin-1 responses via stimulation by different forms of *C. albicans*, we determined cytokine secretion by CD4^+^ T cells cultured with bone marrow-derived dendritic cells (BMDCs) in the presence of anti-CD3 (for T cell stimulation) and either yeast or hyphal *C. albicans* (for BMDC stimulation). As reflected in fractions of positive cells, the yeast form induced substantial IL-17 production—as noted in previous studies of cytokine responses to fungal infection [17]—whereas, in contrast, the hyphal form induced very little IL-17, instead inducing a large amount of IL-10 (Figure 1A). These results correlated with cytokine and master transcriptional regulator mRNA expression accompanying these forms of stimulation (Figure 1B and Figure S1). Because of their unique manner of induction, we called these Dectin1-induced IL-10-producing T cells Dect-10 T cells, anticipating further studies distinguishing these cells from previously defined regulatory T cells (see below).

In further studies of the IL-10-producing cells we assessed cytokine expression by T cells co-cultured with BMDCs in the presence of Dectin-1 ligand (Zymosan Depleted, ZD) and/or a TLR2 ligand (FSL-1). We found that a large fraction of the T cells differentiated into IL-10-producing cells when co-cultured with ZD-stimulated BMDCs (as in the case with hyphal stimulation) whereas very few T cells exhibited such differentiation when co-cultured with FSL-1-stimulated BMDCs (Figure 1C). This suggested that the hyphal cell wall is composed of components favoring Dectin-1 rather than TLR stimulation, whereas the yeast cell wall is composed of components favoring TLR stimulation. Studies of cytokine protein secretion verified these findings and also revealed that ZD (Dectin-1)-stimulated DCs induce modest T cell production of IL-4 prior to IL-10 production (Figure 1D); in addition, ZD-stimulated DCs induce T cells production of IFN-γ at levels equal to that seen with TLR2 stimulation (Figure 1D).

Expanding on these studies of TLR induction, we next stimulated wild-type (WT) BMDCs with intact or fragmented hyphae, with the latter to expose normally shielded TLR ligands in the hyphae cell wall [18], and found that the fragmented hyphae as compared to intact hyphae induced decreased T cell IL-10 and IL-4 production. However, this difference was abolished in T cells similarly stimulated by MyD88^-/-^ BMDCs (Figure 1E,F), and the stimulation of MyD88^-/-^ BMDCs with yeast induced T cells to produce increased amounts of IL-10 compared to stimulation of WT BMDCs (Figure 1C,D). These studies thus suggested that MyD88-pathway signaling induced by TLR ligands expressed by fragmented hyphae or yeast causes the inhibition of IL-10 production, as in the case of direct stimulation with TLR ligands. Finally, inasmuch as α-mannans detected by Dectin-2 are also major carbohydrates composing the cell wall of *C. albicans*, we analyzed Dectin-1^-/-^ and Dectin-2^-/-^ BMDCs-stimulated T cells in the presence of *C. albicans* and found that both Dectin-1- and -2-deficient cells retained their ability to induce IL-10 production when stimulated by *C. albicans* hyphae (Figure S2A,B). This suggested that the two carbohydrate receptors activate common downstream pathways with respect to IL-10 production.

The studies above showing that Dect-10 T cells are induced by DCs stimulated by Dectin1 ligands, but not TLR ligands, suggested that Dect-10 T cells comprise a subset of regulatory T cells that are distinct from other regulatory T cell subsets. To investigate this possibility, we next conducted studies to distinguish these cells from Tr1 cells and Foxp3^+^ Treg cells, i.e., other IL-10-producing regulatory T cell subsets. With respect to Tr1 cells, cells lacking a definitive cell surface marker, we reasoned that identity could be tested by whether Dect-10 T cell development depended on the secretion of IL-27, a STAT3-activating cytokine shown to induce Tr1 cells [19]. With this in mind, we took advantage of the fact that IL-27 is a heterodimeric molecule in which one of its constitutive chains consists of EBI3 [12] and thus determined IL-10 production in co-cultures of WT or EBI3^-/-^ ZD-stimulated BMDCs and WT or EBI3^-/-^ CD4^+^ T cells, i.e., in cultures in which either the DCs or the T cells lack the capacity to produce IL-27. We found that the fraction of cells producing IL-10 in cultures in which either the DCs or the T cells consisted of EBI3^-/-^ cells was substantially the same as the fraction of cells producing IL-10 in cultures containing WT cells (Figure S3A). Thus, Dect-10 T cell development was not IL-27-dependent and these cells were distinct from Tr1 cells. Another critical transcription factor, c-Maf, did not show difference in its transcriptional level between yeast and hyphal *C. albicans* stimulation at the peak time point of IL-10 production, which suggests a negligible contribution to Dect-10 T cell characteristics (Figure S1). With respect to distinguishing Dect-10 cells from Foxp3^+^ Treg T cells, we utilized Foxp3 itself as an identifying marker. Here we determined that co-cultures of ZD- or *C. albicans* hyphae-stimulated BMDCs with CD4^+^ T cells induced large numbers of IL-10-producing cells, i.e., Dect-10 T cells, that did not exhibit Foxp3 expression (Figure S3B). Thus, Dect-10 T cells can, in fact, be distinguished from both Tr1 T cells and Foxp3^+^ Treg T cells. Further studies described below showing that Dect-10 T cells are also distinct from Th2 T cells, as well as studies establishing the distinct IL-10 transcriptional profile of Dect-10 T cells, completed the picture and allowed the conclusion that Dect-10 T cells are in fact a unique subset of IL-10-producing T cells.

### 2.2. High IL-10 Production by Dect-10 Cells Requires T Cell IL-4 Production

As mentioned, Dect-10 T cells produce low levels of IL-4. Thus, in initial studies exploring the molecular mechanisms underlying Dect-10 T cell differentiation, we determined whether IL-4 stimulation was necessary for such differentiation. To this end, we determined Dect-10 T cell induction in populations of IL-4- and STAT6-deficient T cells as well as T cells with conditional deficiency of GATA3 due to the presence of floxed GATA3 under a tamoxifen (estrogen receptor)-regulated Cre activity-. We found that T cells with each of these deficiencies could not be induced to produce IL-10 (or IL-4) by Dectin-1-stimulated BMDC (Figure 2A–C).

In related studies, in this case based on the assumption that Dectin-1-stimulated DC’s act through humoral factors to induce Dect-10 T cells, we assessed the induction of IL-10-producing T cells in cultures of CD4^+^ WT T cells stimulated by plate-bound anti-CD3 in the presence of supernatants from co-cultures of Dectin-1 (ZD)-stimulated BMDC and anti-CD3-stimulated WT or IL-4^-/-^ T cells (Figure 2D). We found that cultures of WT CD4^+^ T cells exposed to supernatants from co-cultures containing WT T cells led to a substantial percentage of cells producing IL-10 and, moreover, that this percentage is augmented by the addition of exogenous rIL-4 to the culture (Figure 2E). In contrast, WT CD4^+^ T cells exposed to supernatants from co-cultures of ZD-stimulated BMDC and IL-4^-/-^ T cells led to a very low percentage of cells producing IL-10 unless exogenous rIL-4 is added to the culture (Figure 2E). Taken together, these studies support the idea that Dectin-1-stimulated DCs do indeed induce Dect-10 T cells via secreted factors, with the latter including IL-4 produced by the Dect-10 T cells themselves.

### 2.3. Gene Profiling Establishes That Dect-10 T Cells Exhibit C/EBPβ Activation

To further investigate the induction of Dect-10 T cells, we conducted microarray studies to compare the transcription profiles of these cells with those of Th0 cells, Th2 cells or Tr1 cells. Correlation analysis of whole-transcriptome gene expression revealed that the global gene expression pattern in Dect-10 T cells was quite distinct from that of the other indicated cell sub-types (Figure 3A). Furthermore, gene function annotation analysis of the most differentially expressed genes using Ingenuity Pathway Analysis (IPA) revealed that Dect-10 T cells, but not Th2 cells or Tr1 cells, expressed genes consistent with C/EBPβ activation and signaling as an “Upstream Regulator” (Figure 3B,C and Tables S1 and S2). To verify this finding, we conducted studies of C/EBPβ^-/-^ T cells to determine if such cells exhibit reduced IL-10 production when stimulated by Dectin-1-activated DCs as described above. We found that C/EBPβ^-/-^ T cells do, in fact, exhibit greatly decreased levels of differentiation into IL-10-producing cells as compared to WT cells (Figure 3D) and thus established that this transcription factor is indeed involved in Dect-10 T cell differentiation.

### 2.4. C/EBPβ Exerts a Positive Effect on Dect-10 T Cell IL-10 Production via Its LIP Isoform

It is well known that C/EBPβ exists as one of three translational isoforms, LAP*, LAP and LIP, the latter having the lowest molecular weight [20]. We therefore investigated which of these isoforms was active in relation to Dect-10 T cell induction. In initial studies we examined the expression level of each of these isoforms in Th0 cells, Tr1 cells, Th2 cells and Dect-10 T cells. While all subsets expressed the LAP isoform, only the Th2 and Dect-10 T cell subsets expressed the LIP isoform, and the concentration of the latter was far higher in Dect-10 T cells than in Th2 T cells (Figure 4A). In further studies, we examined the functional effect of LIP vs. LAP on IL-10 production in C/EBPβ^-/-^ CD4^+^ T cells transduced with a LIP- or LAP-expressing retroviral plasmid. We found that LAP transduction led to reduced percentage of IL-10-positive T cells, whereas LIP transduction led to increased percentage of IL-10-positive T cells compared to control un-transduced cells (Figure 4B). Collectively, these data strongly suggested that the LIP rather than the LAP isoform of C/EBPβ is driving IL-10 production in Dect-10 T cells.

In further studies along these lines, we sought to define how LIP vs. LAP contributes to IL-10 transcription (along with other factors) using HEK293T cells transfected with an IL-10-promoter–luciferase reporter construct. A partial map of the IL-10 promoter based on TRANSFAC screening and previous findings [5,21] shows that the IL-10 promoter region contains two GATA3 binding sites and a C/EBPβ binding site adjacent to a CREB binding site (Figure 4C). We therefore co-transfected the reporter plasmid with plasmids expressing LAP, LIP, GATA3 and CREB in combination with dibutyryl c-AMP (dbc-AMP) to induce the activation of CREB via PKA signaling. We found that luciferase activity generated by the reporter plasmid was greatest when LIP and CREB were co-expressed, particularly when in combination with GATA3 (Figure 4D). Further studies in which HEK293T cells were transfected with truncated mutants of the IL-10-promoter–luciferase reporter construct disclosed that maximum transcription effect requires the presence of the CREB binding site (Figure 4E and Figure S4).

In complementary studies of the deletional mutants of the IL-10-promoter luciferase reporter construct to gain insight into the significance of GATA3 binding, we found that deletion of the distal GATA3 binding site (1.2 kbp upstream of the TSS) led to a very considerable decrease in promoter activity, but not by the proximal GATA3 binding site, suggesting that GATA3 acts as a transcription factor when binding to the distal site; however, such transcription is dependent on concomitant C/EBPβ and/or CREB binding (Figure 4E and Figure S4). The binding of GATA3 to this proximal site could be necessary for epigenetic modification of the physiologic promoter since in studies of the IL-10 promoter in Th2 cells and in studies here showing that cells induced under Dect-10 T cell-inducing conditions but lacking GATA3 exhibit greatly reduced IL-10 expression (Figure S5A,B) as well as reduced acetyl histone 3 binding to the IL-10 promoter, a marker of epigenetic modification of the promoter [5] (Figure S5C).

### 2.5. Regulation of C/EBPβ Isoform Expression in Dect-10 T Cells by mTOR

It has been shown previously that the expression of the LIP or LAP isoforms of C/EBPβ is regulated by the ribosome activity within C/EBPβ mRNA that recognizes a LIP- or LAP-translation-initiating codon. LIP translation occurs when ribosome recognition is directed toward a small upstream open reading frame (uORF) that serves as an LIP regulatory codon [22]. The activation of the mTOR pathway can influence this process by phosphorylating 4EBP1 and thereby releasing a factor (elF-4E) that induces recognition of the uORF [20].

The above facts concerning the C/EBPβ/mTOR interaction led us to determine the effect of blocking the mTOR pathway with rapamycin on LIP and LAP expression and thereby on induction of Dect-10 T cells. In these studies, we showed that the addition of rapamycin to cultures of developing Dect-10 T cells led to greatly decreased LIP expression with little or no effect on LAP expression (Figure 5A). Furthermore, such addition led to a dose-dependent decrease in Dect-10 T cell induction (Figure 5B). Finally, in complementary studies, we found with ChIP studies that the addition of rapamycin led to the decreased binding of C/EBPβ, phospho-CREB1 and CBP to the IL-10 promoter (Figure 5C). Together, these findings provided strong evidence that increased LIP expression by TORC1 signaling underlies the high level of IL-10 production by Dect-10 T cells. Additional support for this comes from the fact that CD4^+^ T cells cultured with supernatants obtained from hyphae-stimulated cultures express a greatly increased level of phospho-4EBP1 and its upstream activator phospho-Akt and the downstream mTOR factor phospho-S6 (Figure 5D).

### 2.6. The LIP Isoform of C/EBPβ Partners with CREB1 for Binding to the IL-10 Promoter

We next turned our attention to whether C/EBPβ has a direct effect on IL-10 transcription in Dect-10 T cells. In the studies addressing this question we first examined LIP/LAP expression and their relative transcriptional effects on IL-10 transcription in Dect-10 T cells generated by *C. albicans* hyphae as well as in Th2 T cells generated by house dust mite (HDM) extract, a well-defined inducer of Th2 cells [23]. These two cell subsets were studied to contrast the IL-10 transcriptional mechanisms in cell subsets which are similar in that both are, at least in part, dependent on IL-4/GATA3, -but are different with respect to the amounts and ratio of IL-4 and IL-10 induced by their respective stimulants: Dect-10 T cells produce far greater amounts of IL-10 than Th2 cells, whereas Th2 cells produce greater amounts of IL-4 than Dect-10 T cells (Figure 6A).

Study of LIP/LAP expression in Dect-10 T cells and Th2 T cells induced by their respective stimuli in both whole Dect-10 T cell and Th2 cell cultures and also in purified Dect-10 T cells and Th2 cells isolated on the basis of IL-10-GFP expression revealed that, whereas Dect-10 T cells exhibit robust LIP expression, Th2 cells exhibit weak LIP expression (Figure 6B,C). These LIP/LAP differences between Dect-10 T cells and Th2 T cells were accompanied in ChIP assays by more avid GATA3 binding to their respective promoter binding sites in Dect-10 T cells than in Th2 cells, particularly with respect to the proximal Dect-10 T cell promoter site (Figure 6D and Figure S5); in addition, C/EBPβ, phospho-CREB1 and CREB-binding protein (CBP) exhibited greater binding to their respective binding sites in Dect-10 T cells than in Th2 cells. The data from these studies emphasized that Dect-10 T cells utilize LIP rather than LAP in IL-10 transcription whereas Th2 cells do not; as such, Dect-10 T cells and Th2 cells are fundamentally different and distinct with respect to mechanism of IL-10 transcription.

As noted in the analysis of their IL-10 transcription in Dect-10 T cells conducted with an IL-10-promoter–luciferase construct, CREB1 was an important factor in Dect-10 T cell IL-10 expression, but only when expressed in combination with LIP (Figure 4D). In a further study of the role of LIP in IL-10 transcription in Dect-10 T cells we next conducted EMSA assays in HEK293 cells transfected with relevant expression plasmids to determine if CREB1 binds to LIP or LAP at the CREB1 binding site in the IL-10 promoter. We found that CREB1, when expressed alone, binds to its binding site on an EMSA oligo probe representing the IL-10 promoter; in addition, such binding is inhibited by addition of a cold probe and is supershifted by anti-CREB1 antibody. However, CREB1, when expressed together with LIP, binds to the EMSA oligo probe both by itself and as a CREB1/LIP complex that causes decreased EMSA oligo mobility and that is supershifted by anti-C/EBPβ antibody. In contrast, CREB1, when expressed together with LAP, binds to the EMSA oligo probe alone and exhibits only a faint band indicative of CREB1 bound to LAP, as indicated by anti-C/EBPβ supershift (Figure 6E). These studies imply that during IL-10 transcription in Dect-10 T cells, CREB1 and LIP form a transactivating complex at adjacent binding sites on the IL-10 promoter of Dect-10 T cells, whereas CREB1 and LAP are far less able to do so. The formation of such complexes can explain the increased binding of CREB1 and the LIP isoform of C/EBPβ to the IL-10 promoter in Dect-10 T cells generated by DCs stimulated with hyphae as opposed to the IL-10 promoter in Th2 cells generated by DCs stimulated with HDM.

### 2.7. mTOR Activity in Dect-10 T Cells Is Driven by Glutaminolysis

Whereas the above findings established that mTOR activation was central to the induction of IL-10 synthesis in Dect-10 T cells, further explanation was necessary for how such activation was induced. Recent studies have shown that the metabolic profile of a T cell is a major determinant of its subset differentiation pathway [24,25]. Accordingly, we performed metabolome studies to identify a metabolic profile that might be unique to Dect-10 T cells and influence its differentiation [26,27]. Such studies revealed that Dect-10 T cells exhibited a distinct metabolic signature that was clearly different from Th2 and Th0 cells according to principal component analysis (Figure 7A). Among the various metabolic pathways associated with this signature (Figure 7B), the glutamate metabolism pathway was of particular interest due to its known connectivity to the mTOR activation pathway; we therefore conducted further studies to understand its possible relevance to the latter pathway in Dect-10 T cells [28,29,30,31].

It is known that glutamine taken up by T cells is converted into glutamate by a process known as glutaminolysis and is then converted into alpha-ketoglutarate (α-KG); the latter then either enters the TCA cycle for the support of mitochondrial respiration or translocates to the lysosome to induce mTOR activity [28] (Figure 7C). On this basis, we first measured the total glutamine consumption as well as the glutaminolysis-linked oxygen consumption rate (OCR) and ATP production of Dect-10 T cells and Th2 T cells activated by TCR stimulation *in vitro*. While we found no difference between these two subsets with respect to total glutamine consumption, Dect-10 T cells exhibited a significantly lower level of OCR and ATP production than Th2 cells (Figure 7D and Figure S6A,B). This suggested that in Dect-10 T cells, glutaminolysis-generated α-KG is utilized relatively less for mitochondrial respiration and more for other purposes such as mTOR activation than is the case in Th2 cells. To examine this possibility, we next determined mTOR activity in comparable TCR-stimulated Dect-10 T cell and Th2 cell populations by evaluation of mTORC1-dependent S6 phosphorylation and found that, indeed, Dect-10 T cells exhibited far stronger mTOR activation than Th2 cells (Figure 7E) by this measure. That increased mTOR activation was, in fact, glutaminase-1-dependent and important to the generation of Dect-10 T cells, which was then shown by the fact that exposure of these cells to a glutaminase inhibitor, 6-diazo-5-oxo-L-norleucine (DON), inhibited the mTOR generation of C/EBPβ-LIP in Dect-10 T cells (Figure 7F); in addition, DON inhibited the induction of IL-10^+^ T cells in the previously described Dect-10 T cell generation culture system to a proportionally greater degree than it inhibited the induction of IL-10^+^ T cells in HDM-inducing Th2 cell-generating culture system, (Figure 7G). Finally, taking a different approach to establishing the dependence of IL-10 production by Dect-10 T cells on glutaminolysis, we showed that they produced little or no IL-10 when cultured in the absence of glutamine, but did so when a cell-permeable form of α-KG, dimethyl α-KG (DMK), was added to the glutamine-deprived culture (Figure 7H). Taken together, these data strongly suggested that the mTOR pathway and down-stream production of IL-10 is activated in Dect-10 T cells by glutaminolysis and its induction of α-KG.

### 2.8. An ActRIB (ALK4) TGFβ Receptor Family Ligand Produced by Dectin-1 Stimulated DCs Is the Inducer of Dect-10 T Cells

In a final series of studies on the nature of Dect-10 T cells we sought to identify the factor or factors produced by Dectin-1-ligand stimulated DCs and present in culture supernatants that induce Dect-10 T cells. In the likelihood that this factor or factors would be reflected in activated gene arrays present in ZD- or hyphae-stimulated DCs but not control DCs, we first performed RNAseq studies of BMDCs purified in the absence of CD4^+^ T cells to eliminate the confounding effects of mRNA expression attributable to T cell stimulation. This approach was justified by preliminary studies showing that BMDCs cultured in the presence of Dectin1 ligands produce soluble factor(s) that stimulate CD4^+^ T cell production of IL-10 even in the absence of activated T cells (Figure 8A). The RNAseq studies were conducted on paired cultures of cells stimulated with either hyphae or yeast or, alternatively, either ZD or “none” (no stimulation) to identify differentially expressed genes (DEGs) that associate with stimulated cells that secrete a factor or factors that induce IL-10.

In our initial examination of the RNAseq data we found by Venn diagram analysis that hyphae and ZD co-up-regulate almost 400 genes among the ~12,000 total genes upregulated by these stimuli (Figure 8B) and that by PCA analysis the up-regulated gene pools of cells cultured under the various conditions were quite different and distinct (Figure 8C); however, clustering analysis showed that genes upregulated by hyphae and ZD stimulation in the heat map were more similar to each other than genes upregulated by either yeast or “none” stimulation (Figure 8D). In further analysis of the RNAseq data, we identified some 35 differentially expressed genes (DEGS) of which three qualified as possible genes of origin of IL-10-inducing factors, *Il6*, *Il33* and *Inhba* (inhibin beta A) (Table S3). Of these three genes, Inhba was by far the most highly expressed and the only one with gene products that signal cells via a receptor family that has been shown to stimulate glutaminolysis and mTOR activation, the TGFβ superfamily [32,33]. This turned our attention to inhibin beta A as the most likely component of the mediator of Dectin-1-stimulated DC induction of Dect-10 T cells.

Extensive previous study has shown that inhibin beta A is a structural component of two possible signaling molecules: activin A, a homodimer of inhibin beta A, and inhibin A, a monomer of inhibin beta A linked to an “alpha” chain [34]. Since both of these molecules act as TGFβ superfamily ligands causing the activation (or inhibition) of cells via a receptor containing the ActRIB (ALK4) TGFβ receptor chain, we next determined if an inhibitor of ALK4 signaling affected the Dectin-1 ligand induction of Dect-10 T cells. Accordingly, we added vactosertib, a well-characterized and specific inhibitor of ALK4/ALK5 TGFβ superfamily signaling [35], to T cell cultures containing supernatants of Dectin-1-stimulated BMDC/T cells culture supernatants (plus IL-4) and found that such addition does indeed cause strong dose-dependent inhibition of Dect-10 T cell induction (Figure 8E). However, whereas this result provided strong support for the thesis that some form of inhibin beta A acts as a Dect-10 T cell inducer, it was subject to the caveat that vactosertib also inhibits ALK5, the TGF-β superfamily receptor component servicing TGF-β itself. Therefore, before proceeding further, we addressed this caveat with a study in which we determined if anti-TGF-β capable of neutralizing all three isoforms of TGF-β to cultures containing supernatants of Dectin-1 ligand-stimulated DCs plus IL_4 (as in studies described above) inhibits Dect-10 T cell induction. We found that such inhibition had no effect on IL-10 production by the Dect-10 T cells induced in the cultures and it was therefore highly unlikely that TGF-β, acting through ALK5, was participating in the Dect-10 T cell inductive pathway (Figure S7). Whether another ALK5 ligand acts in this capacity is also unlikely in this context, but definitive proof that it does not do so awaits additional study.

With the above information in hand, we next considered the possibility that activin A was the inhibin beta A-containing inducer of Dect-10 T cells, as suggested by a prior study showing that activin A signaling cells via a TGFβ superfamily receptor containing an ActRIB (ALK4) chain induces T cell IL-10 production [36]. In studies to examine this possibility we found that cultural supernatants of ZD- or hyphae-stimulated BMDCs did in fact contain higher levels of activin A (identified with an anti-activin A ELISA assay) than supernatants of yeast-stimulated or unstimulated BMDCs; however, it should be noted that the assay could also be identifying inhibin A since the latter also contains an inhibin beta A component. In further studies to determine whether activin A was the Dect-10 T cell-inducing factor, we added follistatin-resistant activin A (inhibin beta A dimer) or anti-activin A to T cell cultures containing supernatants of Dectin-stimulated BMDC/T cells culture supernatants (plus IL-4) that induce Dect-10 T cells as described above; however, we found that such additions neither increased nor decreased (respectively) baseline induction of Dect-10 T cells. Moreover, we found that the addition of a naturally occurring inhibitor of activin A, follistatin [36], or a activin A trap (ActRII-Fc), sotatercept, to T cell cultures containing supernatants of Dectin-1-stimulated BMDC/T cell culture supernatants also failed to change the baseline induction of Dect-10 T cells. Together, these studies strongly suggested that activin A was not an inducer of Dect-10 T cells.

The elimination of activin A as a TGFβ superfamily ligand inducer of Dect-10 T cells leaves open the possibility that inhibin beta A, most likely in the form of a monomer linked to an as yet undefined chain produced by the Dectin-1-stimulated DC is the Dect-10 T cell inducer. Efforts to support this idea with studies showing that the addition of commercially available purified inhibin A to cultures of T cells containing IL-4 augments IL-10 production were not consistently positive, possibly because in this case the added inhibin beta A was not linked to the correct molecular partner. Thus, while it is now possible to conclude that Dect-10 T cells result from TGFβ superfamily signaling, the question of whether the instigator of such signaling is monomer inhibin beta A bound to a chain other than alpha chain requires further study.

### 2.9. Dect-10 T Cells Act as Regulatory T Cells in Renal C. albicans Infection

The fact that Dect-10 T cells produce IL-10 identifies these cells as potential regulatory cells. To verify this, we first determined if Dect-10 T cells regulate *C. albicans* infection, i.e., a condition in which they are likely to be induced via Dectin-1 signaling. In initial studies we sought to show that IL-10-producing Dect-10 T cells occur preferentially at sites of infection where *C. albicans* accumulates in its hyphal form rather than its yeast form, i.e., sites that enable Dect-10 T cell induction. For this, we infected mice with a mutant *C. albicans* strain (Δcph/Δefg) that is locked into the yeast form and cannot produce hyphae [37] and showed that such mice exhibit reduced mortality compared to mice infected with a WT *C. albicans* strain (SC5314) (Figure 9A); as expected, this correlated with reduced fungal burden and inflammation of the kidney, the latter being a lethal feature of hyphal but not yeast *C. albicans* infection (Figure 9B,C). In further studies we showed that CD4^+^ T cells accumulating in the kidneys of mice infected with WT *C. albicans* contained a greater fraction of cells producing IL-10 as compared to mice infected with Δcph/Δefg-*C. albicans* (Figure 9D,E); in addition, Dectin-1^+^ cells isolated from the kidney of WT *C. albicans*-infected mice had an enhanced ability to induce IL-10^+^ cells than similar cells isolated from the spleen (Figure 9F). These studies thus show that IL-10-producing T cells that are likely to be Dect-10 T cells given their association with hyphae-associated ligand, do indeed accumulate at sites of infection at which *C. albicans* is present in its hyphal form.

To determine if the accumulation of Dect-10 T cells in the kidney during *C. albicans* infection affects the survival of infected mice, we next conducted studies comparing infection in IL-10^flox/flox^ and IL-10^flox/flox^-CD4-Cre mice, with the latter mice bearing T cells that cannot express IL-10. In this case we found that a lack of T cells producing IL-10 led to better survival and to decreased fungal burden in the kidney (Figure 9G,H). As shown *in vitro*, this is likely due, in part, to Dect-10 T cell inhibition of the NLRP3 inflammasome and IL-1β/IL-18 production, major effector cytokines mediating *C. albicans* renal inflammation, and protective immune responses [38] (Figure 9I). These studies thus show that IL-10-producing cells likely to be Dect-10 T cells do indeed moderate the immune effector response to severe fungal infection, in this case to the detriment of host survival.

### 2.10. Regulation of Experimental Asthma in Mice by Dect-10 T Cells

Studies in humans and in murine models have shown that exposure to β-glucan can increase or decrease risk for the development or severity of allergic asthma [34]. It was therefore of interest to explore the regulatory effect of Dect-10 T cells on experimental asthma in mice. In initial studies, asthma induced in mice by intra-nasal house dust mite extract (HDM) administration accompanied by ZD administration was evaluated according to the regimen depicted in Figure S8A. This study showed that ZD-induced Dectin-1 stimulation during asthma induction in this model led to significant decreases in the numbers of total cells, CD4^+^ T cells and eosinophils in broncho-alveolar lavage (BAL) fluid, but no change in BAL neutrophils or macrophages (Figure S8B). In addition, ZD administration led to significant decreases in plasma total IgE and HDM-specific IgE (Figure S8C). These decreases in markers of allergic inflammation were, however, counter-balanced by the fact that the fraction of CD4^+^ T cells expressing a variety of Th2 and Th1 cytokines was either unchanged (in BAL) or increased (lung tissue) (Figure S8D). Finally, histological examination of PAS-stained lung tissue disclosed that ZD-treated mice exhibited greatly reduced peri-bronchial cell infiltration and reduced goblet cell hyperplasia (Figure S8E). These findings indicate that whereas total Dectin-1 responses down-regulate key elements of allergic asthma (CD4^+^ T cells, eosinophils and IgE), it does not have a global down-regulatory effect on all aspects of the inflammation.

It is possible that the somewhat incomplete regulatory response in the model studied above is due to the fact that Dectin-1 ligand-mediated DC responses, such as those elicited in this model, encompass effector cell responses as well as regulatory responses. To examine this possibility, we conducted further studies of the regulatory function of Dectin-1-induced Dect-10 T cells in experimental asthma, but in this case determined the role of Dect-10 T cell regulation in the absence of Dectin-1 ligand-mediated effector function by treating the mice with experimental asthma through the administration of Dect-10 T cells previously induced *in vitro*, as shown in the protocol depicted in Figure S9A,B. Here, we found that such administration resulted in reductions in total BAL cells as well as BAL eosinophils, macrophages and CD4^+^ T cells (Figure S9C). In addition, CD4^+^ T cells in both BAL and lung tissue exhibited changes in cytokine expression consistent with a decreased allergic inflammatory response, and plasma IgE levels were greatly decreased (Figure S9D,E). Finally, histologic examination of PAS-stained lung tissue showed that Dect-10 T cell administration led to decreased peri-bronchial cell infiltration and goblet cell hyperplasia (Figure S9F). Thus, overall, this study provides strong evidence that the Dect-10 T cell component of Dectin-1 stimulation mediates the down-regulation of experimental asthma inflammation.

## 3. Discussion

IL-10 was initially categorized as a Th2-type cytokine because it was produced by T cells that also produce IL-4, but in reality, it is produced by a broad range of T cells that are not Th2 T cells, including those producing relatively large amounts of IL-10 in the absence of IL-4 that are known as Tr1 regulatory T cells [3]. Interestingly, the mechanism underlying IL-10 production in the various T cells, despite the fact that the same gene is being activated to produce IL-10, can be T cell type-specific both with respect to mode of induction and method of transcription. In Tr1 regulatory T cells, for instance, IL-10 production is governed by the IL-27 induction of a trio of transcription factors—MAF, IRF4 and BATF—that activate IL-10 transcription only in Tr1 cells [11,39]. In the present study, we describe regulatory IL-10-producing T cells induced by a soluble factor produced by Dectin-1 ligand-stimulated DCs acting via TGFβ family receptor signaling that undergo a hitherto undescribed IL-10-producing transcriptional process unique to these IL-10-producing regulatory T cells. Given the differences between these cells and Tr1 cells and, indeed, other IL-10-producing regulatory T cells, it seems appropriate to refer to these cells by a unique name, Dect-10 T cells.

The transcriptional mechanism underlying high-level IL-10 differentiation in Dect-10 T cells was found to be characterized by two interlocking molecular processes. The first involves the dendritic cell/anti-CD3 monoclonal antibody stimulation of nascent Dect-10 T cell production of IL-4 and resultant auto-activation of STAT6 and GATA3. The critical element in this signaling event is most likely GATA3 activation since we could show that GATA3 interacts with the Dect-10 T cell IL-10 promoter at two sites: a distal binding site where GATA3 acts as a direct transcription factor and at a proximal non-binding site where it acts indirectly on transcription as an epigenetic factor that augments histone acetylation [5]. Such GATA3 activity differs from that in Th2 cells since it has been shown that GATA3 does not act as a direct transcription factor for IL-10 in developing Th2 cells [5].

The second process guiding the differentiation of Dect-10 T cells consists of the activation of the TORC1 arm of the mTOR signaling pathway. This was initially suggested by the fact that in gene profiling studies we showed that Dect-10 T cell differentiation—as opposed to Tr1 and Th2 cell differentiation—involve C/EBPβ transactivation. Together with the subsequent studies showing that the LIP rather than the LAP isoform of C/EBPβ was necessary for IL-10 synthesis in Dect-10 T cells, pointed to TORC1 activation which is necessary for LIP isoform generation [20]. Ultimately, we verified this possibility through studies showing that IL-10 production in Dect-10 T cells is subject to dose-dependent inhibition by rapamycin, an inhibitor of TORC1 signaling, and such inhibition correlates with loss of LIP expression. Interestingly, Th2 cells express little or no LIP and the presence of latter therefore serves to emphasize the distinction between these two IL-10-producing cell subtypes at the molecular level.

Having established that TORC1 activation was a component of IL-10 induction in Dect-10 T cells, it became of interest to define the nature of the stimulus that induced such activation. For this, we turned to metabolome analysis of Dect-10 T cells on the basis of previous studies showing that T cell subset differentiation is greatly influenced by metabolic factors [24]. Indeed, upon subjecting Dect-10 T cells to such analysis, we found that these cells had a metabolic profile that was distinct from that characterizing Th0 and Th2 cells, and one of the most highly up-regulated metabolic pathways was the glutamine metabolism pathway, i.e., a pathway that had been shown previously to lead to the activation of mTORC1 via its generation of alpha-ketoglutarate (α-KG). Evidence that this pathway was, in fact, involved in IL-10 production in Dect-10 T cells came from subsequent studies showing that inhibition of glutaminase-1—an enzyme important in the initiation of glutaminolysis—blocks LIP expression in Dect-10 T cells, indicating that the inhibition was due, at least in part, to down-regulation of mTORC1 function; in addition, such inhibition down-regulated IL-10 production in Dect-10 T cells to a far greater extent than in Th2 cells, i.e., cells in which IL-10 production is independent of mTORC1 function and LIP generation). These findings provided clear evidence that the mTORC1 activation and LIP generation driving IL-10 transcription in Dect-10 T cells, discussed above, is dependent on glutaminolysis. Additional evidence along these lines came from studies showing that Dect-10 T cells failed to become IL-10-expressing cells when cultured in a glutamine-deprived medium, although such expression was restored upon addition of a cell-penetrable form of α-KG This latter observation fits well with prior studies showing that amino acid-mediated mTORC1 activation is dependent on at least two GTPase-related mechanisms, both of which are activated by α-KG [40,41].

Glutamine metabolism has previously been shown to play a multifaceted role in the differentiation of T cells into IL-17-producing cells, i.e., Th17 cells [42]. In studies probing the contribution of glutaminolysis to such glutamine metabolism-supported differentiation, it was shown that genetic deletion or inhibition of glutaminase-1, the major initiator of glutaminolysis, inhibits Th17 differentiation, but not the differentiation of other T cell subsets [30,43]. Thus, Dect-10 T cells and Th17 T cells are similar to each other and distinct from other T cell subsets (including Th2 cells) with respect to dependence on glutaminase-1-initiated glutaminolysis. However, the outcome of glutaminolysis in developing Th17 T cells and Dect-10 T cells is vastly different in that, whereas cell-penetrable α-KG rescues IL-10 production of developing Dect-10 T cells cultured in the absence of glutamine (as shown here), it does not rescue IL-17 production by developing Th17 cells cultured under similar conditions [30]. Th17 differentiation cultured in the absence of glutamine is, however, rescued by cell-penetrable 2-hydroxyglutarate (2-HG), the latter being a direct metabolite of α-KG that favors Th17 development, not by activation of mTORC1, but rather by acting epigenetically to inhibit Foxp3 transcription and/or by stabilizing HIF-1α, an enhancer of RORγt [44]. It should be noted, however, that glutamine-related activation of mTORC1 does participate in Th17 differentiation through several non-glutaminolysis-mediated mechanisms, including facilitation of leucine transport and leucine-mediated mTORC1 activation [29,42].

Whereas glutaminolysis has not been shown to mediate IL-10 synthesis in regulatory T cells prior to the present study, there is a recent report that it does so in regulatory B cells [45]. In this report it was demonstrated that regulatory B10^+^ B cells producing high levels of IL-10 exhibit increased expression of a glutamine transporter, ASCT2, and required glutamine for IL-10 production. In addition, IL-10 production and IL-10^+^ B cell numbers were reduced by exposure to a glutaminase-1 inhibitor and restored by the provision of α-KG, pointing to glutaminolysis activation of mTORC1 as the cause of the high level of IL-10 expression. This proved to be the case since the culture of B cells in the presence of rapamycin, as in the present study, greatly inhibited IL-10^+^ B cell numbers and IL-10 production. The glutaminolysis-mTORC1-mediated augmentation of IL-10 production in this case was attributed to activated mTORC1 inhibition of GSK3β, since the latter had been shown in prior studies to inhibit IL-10 production in DCs [46], and in this study it was shown that provision of a GSK3β inhibitor restored IL-10 production in B cells treated with rapamycin. However, this explanation of the enhanced IL-10 production in a B cell leaves open the question of how GSK3β inhibition affects IL-10 synthesis on a mechanistic level. One possibility, in view of the studies reported here, is that GSK3β limits the ability of high-IL-10-producing B cells to form C/EBPβ/CREB complexes.

The elucidation of the intracellular events leading to IL-10 transcription in Dect-10 T cells discussed above cleared the way for studies defining the extracellular stimulus that set these events in motion. Initial studies showing that these cells can be induced by supernatants derived from Dectin-1 ligand-stimulated DC/activated T cell co-cultures, or more simply from Dectin1-ligand-stimulated DC cultures, established that the inducing stimulus was a soluble factor secreted by the Dectin-1 ligand-stimulated DCs; thus, our studies were designed to identify that factor. Based on RNAseq studies that identified Inhba as the most prominent differentially expressed gene in DCs stimulated by Dectin-1 ligands that induce secretion of Dect-10 T cell-inducing factors, and that the Inhba gene product inhibin beta A expressed in a dimeric form as activin or in another molecular form was likely to be signaling cells via the TGFβ superfamily of receptors, we determined whether Vactosertib, an ALK4/ALK5 TGFβRI receptor inhibitor, could block Dect-10 T cell induction when added to T cells cultured with supernatant of Dectin-1-stimulated DCs. Vactosertib did in fact block such induction, indicating that the inducing factor was indeed among the relatively limited number of molecules that signal cells via one of the TGFβ superfamily receptors.

As part of a broad study of the possible ligands for TGFβ family receptors that can act as Dect-10 T cell inducers, we investigated whether TGFβ itself was the inducing factor, given the fact that *Tgfb1* was also a differentially expressed gene in ZD vs. “none” RNAseq studies and that TGFβ contributes both to the induction of Foxp3^+^ Tregs and Tr1 T cell induction, both cell types that exhibit high levels of IL-10 production [12,47]. This initial possibility, however, was proven untenable by repeated studies showing that addition of anti-TGFβ to Dectin-10 T cell inducing cultures failed to inhibit Dect-10 T cell induction.

A second possibility was that activin A was the inducer, since this cytokine also signals cells via a TGFβ superfamily receptor (in this case utilizing ALK4 TGFβRI, mainly) and has been shown to induce regulatory cells—in this case Tr1-like cells [48]. In addition, as alluded to above, this factor is a homodimer of inhibin beta A, a protein that is encoded by the most prominent differentially expressed gene identified in RNAseq studies. This possibility, however, also proved to be untenable, firstly because in prior studies activin A induction of Tr1-like cells was shown to be driven by an IRF4/AhR-mediated mechanism unrelated to mTORC1, and secondly because of studies conducted here showing that anti-activin A does not block Dect-10 T cell induction and that activin A does not augment such induction; in addition, follistatin, a potent natural inhibitor of activin A, and sotatercept, a recently developed Activin A trap, do not block Dect-10 T cell induction.

A third possibility was that the inducer of Dect-10 T cells was inhibin itself, in this case not as a dimer (i.e., as activin A) and not bound to α chain in which case it acts (mainly in studies of reproductive cells) as an inhibitor of activin A signaling by forming a non-signaling complex with β-glucan that blocks activin A binding to its receptor [34]. However, in studies addressing this possibility, we found that the addition of inhibin A linked to alpha chain did not consistently induce IL-10 production in CD4^+^ T cell populations. One possible explanation of these inconsistent results is that the added inhibin A consisted of inhibin beta A linked to alpha chain (i.e., inhibin A) and is thus a molecule that may differ from that being produced by Dectin-1 ligand-stimulated DCs which may be linked to a chain other than alpha chain. Overall, the best conclusion is that Dectin-10 T cells are clearly induced by TGFβ superfamily signaling that is independent of TGFβ and activin and may be the result of novel inhibin beta A signaling, but this is yet to be verified by further study.

To demonstrate that Dect-10 T cells do in fact manifest a function that justifies their designation as regulatory cells, we showed that following infection of mice with *C. albicans* IL-10-producing cells are more abundant at a tissue site at which the hyphal form of the organism capable of inducing these cells is present. Since these are the conditions in which Dect-10 T cells are induced *in vitro*, it was likely that the IL-10-producing T cells accumulating at hyphae-rich sites were, in fact, Dect-10 T cells, but this could not be formally proven in the absence of identifying marker studies. We then showed that infection of mice lacking T cells improved survival, indicating that the presence of Dect-10 T cells causes down-regulation of the T cell effector function and reduced clearance of the organism. In accompanying *in vitro* studies, we showed that Dect-10 T cells down-regulate NLRP3 inflammasome responses, a potent mechanism of *C. albicans* inflammation and protection from infection; thus, the reduced protective response indicated by the mouse survival studies may have been due, in part, to decreased IL-1β and IL-18 production by the NLRP3 inflammasome. It should be noted, however, that fungal infection-associated NLRP3 inflammasome responses can also cause detrimental fungal inflammation [49]; in this hypothetical case, a Dectin-10 T cell response would have a positive outcome.

We also studied the regulatory potential of Dect-10 T cells in a mouse model of asthma induced by house dust mite administration based on the fact that clinical exposure to *C. albicans* or to β-glucan has both negative and positive effects on the severity of asthma [50,51]. We found that the administration of Dectin-1 ligand or the administration of Dect-10 T cells generated *in vitro* to mice with induced asthmatic lung inflammation led to the down-regulation of asthma-inducing responses. The administration of Dect-10 T cells provided a more definitive result, firstly because, in this case, we could be more sure that the effect was due to Dect-10 T cells and secondly because Dect-10 T cell administration had a more complete therapeutic effect, with the latter possibly reflecting the fact that the response to Dectin-1-ligand includes effector cell components that do not contribute to—or may even oppose—asthma amelioration. Overall, these studies established that Dect-10 T cells exert regulatory activity on key aspects of a Th2-type inflammation and explain the inflammation-reducing effects of exposure to fungal antigens in both murine asthma models and in patients with asthma [52]. As such, they correlate with an earlier report showing that Curdlan, a crude Dectin-1 ligand which contains both Dectin-1 and TLR ligands, induces regulatory macrophages that also ameliorate allergic symptoms in mice [53]. Harnessing this positive regulatory response for the clinical treatment of asthma is likely to require the development of methods of eliciting Dect-10 T cell development in the absence of concomitant effector T cell development. This is potentially possible given the fact that in our earliest studies we found that regulatory T cell and effector T cell induction by *C. albicans* was differentially regulated by various *C. albicans* components.

## 4. Materials and Methods

### 4.1. Mice

Specific pathogen-free C57BL/6J, Vert-X (IL-10-GFP), IL-4^-/-^, STAT6^-/-^, MyD88^-/-^, RAG2^-/-^, Foxp3-RFP, CD4-Cre, OT-II TCR Tg mice were purchased from Jackson Laboratories. Dectin-1^-/-^, Dectin-2^-/-^ mice were kind gifts from Dr. Y. Iwakura (Tokyo University of Science). C/EBPβ^-/-^ mice were provided by Dr. P. Johnson (NCI, NIH). IL-10^flox/flox^ mice were kindly provided by Dr. R. Blumberg (Brigham and Woman’s Hospital, Boston). GATA3^flox/flox^/ERT2-Cre mice (Line 8445) were obtained from the NIAID-Taconic repository. IL-10^flox/flox^/CD4-Cre, OT-II/IL-10-GFP, and IL-10-GFP/Foxp3-RFP were bred in the animal facility. All mice used were 6–14 weeks of age. All experiments were carried out under protocols approved by the NIAID Animal Care and Use Committee and animal committee of Foundation for Biomedical Research and Innovation at Kobe and followed the National Institutes of Health Laboratory Animal Care Guidelines.

### 4.2. In Vitro T Cell Differentiation

Naïve CD4^+^ T cells from spleen and lymph nodes were isolated using the CD4^+^ CD62L^+^ T Cell Isolation Kit Il (Miltenyi Biotec, San Diego, CA, USA) according to the manufacturer’s instructions. Naïve CD4^+^ T cells were stimulated by BMDCs with anti-CD3 mAb (clone: 145-2C11, 0.2 μg/mL, eBioscience, San Diego, CA, USA), in the presence of FSL-1 (10 nM, Invivogen, San Diego, CA, USA), Zymosan Depleted *S. cerevisiae* (100 μg/mL, Invivogen), Zymosan (100 μg/mL, Invivogen), heat killed *Candida albicans* (1 × 10^5^/mL), or house dust mite extract (150 U/mL, gift from Dr. D. Metcalfe, NIAID, NIH). For antigen-presenting cell-free naïve CD4^+^ T cell stimulation, cells were added to anti-CD3 mAb-coated plates (2 μg/mL) along with soluble anti-CD28 mAb (clone: 37.51, 2 μg/mL, eBioscience) in the presence of collected supernatants of T cell-BMDC coculture as indicated above. Rapamycin (Cell Signaling Technology, Danvers, MA, USA), 6-Diazo-5-oxo-L-norleucine (DON, Wako, Tokyo, Japan), and dimethyl-α-ketoglutarate (DMK, Tokyo Chemical Industry, Tokyo, Japan), anti-TGFβ (clone: 1D11.16.8, BioXcell, Lebanon, NH, USA) was added using the concentration indicated.

### 4.3. Intracellular Staining

Cells were stimulated with plate-bound anti-CD3 mAb on 96-well flat-bottom plates for 6 h and treated with BD GolgiPlug (BD Biosciences, Franklin Lakes, NJ, USA) for the final 2 h of culture. The stimulated cells were first FcR (Biolegend, San Diego, CA, USA)-blocked, followed by APC-eFluor780 viability dye (Invitrogen, Carlsbad, CA, USA) and PerCpCy5.5-anti-CD4 mAb staining, together with Brilliant Violet (BV) 421-anti-Thy1.1 mAb where indicated. After fixation and permeabilization, cells were stained with FITC-anti-IL-10 mAb, PE-anti-IL-5 mAb, PE-anti-IL-4 mAb, PE-Cy7-anti-IL-4 mAb, APC-anti-IL-13 mAb. APC-anti-phospho-Akt (S473; eBioscience), PE-anti-phospho-4EBP1 (T35/T45; eBioscience), and Alexa647-anti-phospho-S6 (S235/S236; Cell Signaling Technology) were stained according to eBioscience intracellular protein staining protocols. All fluorescence-labeled antibodies were purchased from Biolegend otherwise indicated. Flow cytometry was performed by FACSCanto and FACSFortessa (BD Biosciences) and analyzed with FlowJo software v10.6 (BD Biosciences).

### 4.4. Quantitative PCR

Total RNA was isolated using Tripure (Roche, Mumbai, India) and RNeasy Mini Kit (Qiagen). First strand cDNA synthesis was performed using High Capacity cDNA Reverse Transcription Kit (ABI) according to the manufacturer’s instruction and amplified with a thermal cycler (ABI). cDNA were then processed for qPCR using PowerUp™ SYBR^®^ Green Master Mix (ThermoFisher Scientific, Waltham, MA, USA) with primers indicated in Table S4. Expression for each gene was indicated as relative increase against b-actin.

### 4.5. ELISA

Culture supernatants were stored in -20 degrees upon harvest. IL-10 (Mouse IL-10 ELISA Set, BD Biosciences), IL-4 (Mouse IL-4 ELISA Set, BD Biosciences), IFN-γ (Mouse IFN-γ ELISA Set, BD Biosciences), IL-1β (Mouse IL-1β ELISA Set, BD Biosciences), glutamine (Glutamine Assay Kit-WST, Dojindo, Munich, Germany) levels were measured according to the manufacturer’s instructions.

### 4.6. Western Blotting

HEK293T cells were transfected with the indicated expression plasmid using X-tremGENE HD (Roche). Whole cell lysates were prepared with RIPA buffer (Santa Cruz Biotechnology, Santa Cruz, CA, USA) with cOmplete Mini Protease Inhibitor Cocktail and PhosSTOP Phosphatase Inhibitor Cocktail (Roche). The samples were incubated with LDS sample buffer and reducing reagent (Life Technologies, Shanghai, China) for 10 min at 70 °C and subjected to SDS-PAGE. After electrophoresis, the proteins were transferred to nitrocellulose membrane and then blocked with BSA; the membrane was then immunoblotted with the indicated primary antibodies, followed by horseradish peroxidase-conjugated secondary antibodies. The signals were developed by WesternBright™ Sirius™ HRP Substrate (Advansta, San Jose, CA, USA) and detected using Omega Lum™ G Imaging System (Aplegen, San Francisco, CA, USA). Nuclear fraction was extracted by NE-PER Nuclear and Cytoplasmic Extraction Kit (Pierce, Woodland Hills, CA, USA) according to manufacturer’s protocol with slight modification using RIPA buffer and pulse sonication after cytoplasmic fraction isolation. For immunoprecipitation, the lysates were incubated with anti-FLAG mAb (M2, Sigma, Kawasaki-shi, Japan)-conjugated Dynabeads Protein G for 1 h in 4 °C; after washing the beads three times with lysis buffer, the beads were processed for elution using 50 mM Glycine (pH = 2). The following antibodies were used for blotting; C/EBPβ (clone: E299, Abcam, Cambridge, UK), phospho-CREB1 (clone: 87G3, Cell Signaling Technology), CREB1 (clone: 48H2, Cell Signaling Technology), GATA3 (clone: HG3-31 or D16, Santa Cruz), and a-Tubulin (clone: TU-02, Santa Cruz).

### 4.7. Bone Marrow-Derived Dendritic Cells (BMDCs) and Macrophages (BMDMs) Differentiation

For BMDCs, bone marrow cells from the femurs were cultured in the presence of recombinant mouse GM-CSF (20 ng/mL, Peprotech, Rocky Hill, NJ, USA) for 6 days, supplemented with fresh media on day 2 and 4. On day 6, the cells were replaced with fresh media without GM-CSF, and loosely attached cells were collected for use on day 7. For BMDMs, bone marrow cells were cultured in the presence of M-CSF (20 ng/mL, Peprotech), replenished with fresh media on day 3. BMDMs were used on day 7. For the analysis of IL-1β production, BMDMs were first primed with LPS (200 ng/mL) and then added ATP (5 mM) for 24 h. Culture supernatant was collected for ELISA studies.

### 4.8. Retroviral Transduction

Mouse C/EBPβ retroviral plasmids were generated by inserting C/EBPβ cDNAs into MSCV-IRES-Thy1.1 DEST (Addgene plasmid #17442). Plat-E cells were transfected by XtremeGENE HP (Roche) with retroviral plasmids, and supernatants were collected after 48 and 72 h. The virus supernatants were added to a Retronectin (Clonetech, San Jose, CA, USA) and anti-CD3 mAb-coated plates and centrifuged for 2 h in 32 °C. After washing the wells with PBS, naïve CD4^+^ T cells were added and stimulated with soluble anti-CD28 mAb as indicated.

### 4.9. Reporter Assay

IL-10 promoter plasmid was cloned using mouse genomic DNA as a template. The PCR products obtained were inserted into pGL4.15 luciferase vector (Promega, Madison, WI, USA). Deletion mutations were introduced by using a QuickChange II site-directed mutagenesis kit (Stratagene, La Jolla, CA, USA). All constructs were verified by Sanger sequencing. HEK293T cells were transfected with the luciferase reporter construct, pGL4.74-hRluc (Promega), and indicated expression plasmids. Luciferase activity was measured by using a dual luciferase assay system (Promega). Each condition was performed in duplicated fashion.

### 4.10. ChIP-qPCR

Cells were fixed in 1% formaldehyde for 10 min at room temperature. Cells were then sonicated using Sonifier 250 (Branson, Branson, MO, USA) and approximately 300 to 500 bp of sheared DNA were incubated overnight at 4 °C with 1 μg of anti H3K27Ac mAb (ab4729, Abcam), anti-C/EBPβ (C-19, Santa Cruz), anti-CREB1 (clone: 48H2, Cell Signaling Technology), anti-GATA3 (clone: HG3-31, Santa Cruz), anti-CBP (C15410224, Diagenode, Denville, NJ, USA) or corresponding isotype control antibodies. The chromatin was immunoprecipiated using Dynabeads Protein G (ThermoFisher Scientific) and processed for quantitative PCR using primers indicated in Supplementary Materials. The levels of ChIP DNA were normalized against input DNA.

### 4.11. Microarray Expression Analysis

Total RNA was isolated from 4 different cell culture preparations each in duplicate (*n* = 2 biological replicates). Naïve CD4^+^ T cells were isolated from the spleen and lymph nodes of C57BL/6J mice by MACS separation techniques. These cells were stimulated by bone marrow derived-dendritic cells (BMDC) in the presence of anti-CD3 mAb (0.2 μg/mL) with or without Zymosan Depleted. Three days later, these culture supernatants were collected and added into a culture where naïve CD4^+^ T cells were stimulated with plate-bound anti-CD3 mAb and soluble anti-CD28 mAb (without BMDCs). The cells were collected at day 5 and processed for RNA isolation using Tripure and RNeasy. For other helper T cell induction, naive CD4^+^ T cells were stimulated by plate-bound anti-CD3 mAb and soluble anti-CD28 mAb in the presence of recombinant-IL-4 (20 ng/mL) and anti-IFN-γ mAb (10 μg/mL) for Th2 cells, and recombinant IL-27 (50 ng/mL) for Tr1 cells, for 5 days and processed for RNA isolation using Trizol (Thermo Fisher Scientific, Carlsbad, CA, USA) and RNeasy (QIAGEN, Hilden, Germany). The quality of extracted RNA was verified by Bioanalyzer and shown to have an RNA Integrity Number (RIN) greater than 9.0 for all samples. The amplification and labeling of the RNA samples were performed using the Illumina TotalPrep RNA Amplification (Applied Biosystems, Carlsbad, CA, USA) and with an input of 500 ng of total RNA per sample. A biotinylated RNA product was hybridized to Illumina MouseRef-8 v2.0 Expression BeadChip (Gene Expression Omnibus accession no. GPL6885), having 25,697 unique probes, using the reagents provided, and imaged using the Illumina HiScan-SQ. Signal data were extracted from the image files with the Gene Expression module (v. 1.9.0) of the GenomeStudio software (v. 2011.1) from Illumina, Inc., San Diego, CA, USA. Signal intensities were converted to log2 scale. The calculation of detection *p*-values is described in the GenomeStudio Gene Expression Module User Guide. Data for array probes with insufficient signal were removed from the dataset (detection was defined as at least two arrays with detection *p*-value < 0.1). After dropping non-performing probes, quantile normalization was applied across all arrays. ANOVA was performed on the normalized log2 expression estimates to test for mRNA expression differences by treatment, with contrasts for Tr1, Th2 or Dect-10 T cells against the Th0 cells. A *p*-value of 0.05 was used for the statistical significance cutoff, after adjusting for the False Discovery Rate using the Benjamini–Hochberg method to account for multiple testing. Statistical analysis was performed using JMP/Genomics software version 6.0 (SAS Institute Inc., Cary, NC, USA). The microarray data have been deposited in NCBI’s Gene Expression Omnibus and are accessible through GEO Series accession number GSE87673 (https://www.ncbi.nlm.nih.gov/geo/query/acc.cgi?acc=GSE87673, 16 September 2026).

### 4.12. Pathway Analysis

Upstream regulator predictions were computed in IPA software version 16542223 (Ingenuity, QIAGEN, Redwood City, CA, USA). As input for the IPA, we took, for each ANOVA contrast, the names of 575 microarray probes having the largest absolute expression difference. The search parameter settings were as follows: reference set = Illumina Ref-8 v2.0; species = Mouse; relationships = relaxed; confidence = Experimentally Observed or High Confidence prediction; data source = Ingenuity Expert/ExpertAssist Findings or Gene Ontology. This analysis infers relatively increased or decreased activity of regulatory molecules based on the empirical behavior of their downstream target genes (expression observed by microarray) and the known relationship (as maintained in the IPA database) between the regulator and target. Upstream regulator analysis indicates significance by (1) an overlap *p*-value that tests for statistical enrichment of genes in the input set against the set of known target genes for each regulator, and (2) an activation Z-score, which tests the consistency of activity predictions, based on directional relationships between a regulator and its target genes, with the up/down expression of the target genes measured by microarray. The approach is described in a whitepaper titled “Ingenuity Upstream Regulator Analysis” (http://pages.ingenuity.com/rs/ingenuity/images/0812%20upstream_regulator_analysis_whitepaper.pdf, accessed on 1 October 2013). Bias correction is a statistical adjustment to the Z-scores to compensate for potentially inadequate representation of up-regulated or down-regulated target genes in the database for that regulator. Upstream regulators were selected as “cell-type specific” (Figure 3B) if the bias-corrected Z-score was >2 or ≤2, the overlap *p*-value was <1 × 10^−3^, and the target set was at least 7 genes for the specific cell type, and the Z-scores for other two (non-specific) cell types were between 1 and −1. BCL6 was also included in the list since it was one of the most differential regulators between Dect-10 T cells and Th2.

### 4.13. Transcription Factor Binding Sequence Screening

The identification of transcription factor-binding motifs was performed with TRANSFAC (Biobase International, Jinan, China) using the “vertebrates” database for mouse IL-10 promoter region (−1500 to +100 from transcription start site).

### 4.14. GC-MS Analysis for Metabolome

Mixed solvent (250 µL; MeOH:H_2_O:CHCl3 = 2.5:1:1) was added to cells (2 × 10^6^) and then a 10 µL aliquot containing 0.5 mg/mL Sinapinic acid (internal standard) was added. The samples were sonicated for 20 sec in a sonicator (UR-21P; Tomy Seiko Co., Ltd., Tokyo, Japan), incubated at 37 °C, 1200 rpm for 30 min, and then centrifuged at 15,000× *g* for 3 min at 4 °C. A 200 µL sample of the supernatant, which contains extracted aqueous metabolites, was transferred to a clean 1.5 mL tube and mixed with 200 µL Milli-Q water. The tubes were vortexed and centrifuged at 15,000× *g* for 3 min at 4 °C. Then, a 350 µL amount of each supernatant was transferred to a clear 1.5 mL tube and evaporated in a vacuum centrifuge dryer for 20 min. The samples were subsequently freeze-dried overnight. For oximation, 40 µL methoxyamine hydrochloride (20 mg/mL) in pyridine was added to each sample, followed by vortexing and incubation for 90 min at 30 °C, 1200 rpm. For trimethyl silylation, 20 µL N-methyl-N-TMS-trifluoroacetamide was added to each sample, followed by incubation at 37 °C, 1200 rpm for 30 min and centrifugation at 15,000× *g* for 5 min. The resulting supernatant was analyzed on a GCMS-QP 2010 Ultra (Shimadzu Corp., Kyoto, Japan) using the following parameters. The column was a 30 m × 0.25 mm i.d. fused silica capillary column coated with 0.25 µm CP-SIL 8 CB low-bleed/MS (Agilent Technologies, Santa Clara, CA, USA). The front inlet temperature was 230 °C. The helium gas flow rate through the column was 1.12 mL/min. The column temperature was held at 80 °C for 2 min isothermally, increased by 15 °C/min to 330 °C, and held at 330 °C for 6 min isothermally. The interface and ion source temperatures were 250 °C and 200 °C, respectively. The mass spectrometry (MS) scan started at 3.55 min and ended at 24.00 min with scans recorded over a mass range 85–500 m/z at a scan speed of 10,000 amu/s. Injection volume was 1 µL and split mode was 25/1 (*v*/*v*).

Data were processed using the free software MS-DIAL (RIKEN Center, Yokohama, Japan). MS-DIAL is a vendor-free metabolomics software that supports all data-processing procedures, including spectral centroiding, peak detection followed by the assignment of MS spectrum, metabolite annotation, peak alignment, data normalization, statistical analysis and result export as a common format. PCA (principal component analysis), MSEA (metabolite set enrichment analysis), pathway analysis was performed with MetaboAnalyst 6.0. (http://www.metaboanalyst.ca/, accessed on 2 September 2026). To test for global differences in metabolic profiles across experimental conditions in Figure 7A, Permutational Multivariate Analysis of Variance (PERMANOVA) was performed using 999 permutations. The analysis revealed a statistically significant distinction between groups (F = 12.298, R^2^ = 0.80389, *p* = 0.004), with group classification accounting for 80.4% of the total metabolic variation”.

### 4.15. Measurement of Oxygen Consumption Rates

Oxygen consumption was measured using an XFe96 Extracellular Flux Analyzer (Agilent Technologies, Santa Clara, CA, USA) with an Agilent Seahorse XF Cell Mito Stress Test Kit (Agilent Technologies, Santa Clara, CA, USA) according to manufacturer’s instruction. Briefly, 2 × 10^5^ cells were seeded on Corning Cell-Tak coated XFe96-well culture plate and then processed for oxygen consumption measurement by the sequential addition of glutamine, oligomycin, FCCP, and rotenone/actinomycin A. Glutaminolysis-linked OCR of Th2 and Dect-10 T cells were calculated by subtracting the OCR level of cells cultured in Gln-free media from the Gln (+) cells using the measurement before Oligomycin addition. ATP production rate of Th2 and Dect-10 T cells were calculated by subtracting the value of Gln free- or Gln(+)-OCR measurement as follows: ((Measurement before Oligmoycin injection) − (Measurement before FCCP injection)).

### 4.16. EMSA

FLAG-CREB1, FLAG-CREB1 co-expressed with LAP, or FLAG-CREB1 co-expressed with LIP protein in HEK293T was immunoprecipitated by Dynabeads Protein G conjugated with anti-FLAG antibody (M2, Sigma Aldrich, St. Louis, MI, USA) and eluted by 3x FLAG peptide (Sigma Aldrich). After equalization of the amount of FLAG-CREB1 protein by densitometric analysis (Image J, version 1.50f 3), samples were incubated with biotinylated oligonucleotides. DNA-protein complex was separated by 6% DNA Retardation Gels (ThermoFisher Scientific) and transferred to BrightStar-Plus Positively Charged Nylon Membrane (ThermoFisher Scientific). Signals were developed using Chemiluminescent Nucleic Acid Detection Module Kit (ThermoFisher Scientific) according to the manufacturer’s instruction and detected by Omega Lum™ G Imaging System. The oligonucleotide sequence is as follows: 5′-TGGAGGAAACAATTATTTCTCAATCCTAATATGTTCTGGAATAGCCCATTTATCCACGTCATTATGACCTGGGA-3′.

### 4.17. RNA Sequencing

RNA from BMDC stimulated for 2 days were isolated using ISOGEN with Spin Column kit as per the manufacturer’s instructions (Nippongene, Tokyo, Japan). Isolated RNA was then sequenced by Novogene. Briefly, messenger RNA was purified from total RNA using poly-T oligo-attached magnetic beads. After fragmentation, the first strand cDNA was synthesized using random hexamer primers, followed by the synthesis of the second strand cDNA. Pair-end sequencing was performed using the NovaSeq 6000 (Illumina) platform. The raw reads in FASTQ format were initially processed and filtered using fastp (v0.23.2). In this step, clean reads were obtained by removing reads containing adapters, reads containing poly-N, and low-quality reads from the raw data. Simultaneously, the Q20, Q30, and GC content of the clean data were calculated. All downstream analyses were based on the clean data with high quality. Differential expression analysis between conditions was performed using the DESeq2 R package which provides statistical routines for determining differential expression in digital gene expression data using a model based on the negative binomial distribution. The resulting *p*-values were adjusted using Benjamini and Hochberg’s approach for controlling the False Discovery Rate (FDR). PCA was conducted to test for global differences in transcriptome profiles across experimental conditions in Figure 8C. PERMANOVA using 199 permutations revealed a statistically significant distinction between groups (F = 3668.5, R^2^ = 0.99927, *p* = 0.005), with group classification accounting for 99.93% of the total transcriptomic variation.

### 4.18. Candida albicans Preparation and Infection

*Candida albicans* strains SC5314 and Δcph/Δefg (gifts from Dr. J. Kwon-Chung, NIAID, NIH) were grown in yeast extract, peptone, and dextrose medium containing penicillin and streptomycin (Invitrogen) in a shaking incubator at 30 °C. Cells were centrifuged, washed in PBS, counted using a hemocytometer, and were heat-killed by incubation at 65 °C for 1 h. For transition of SC5314 to hyphae, cells were suspended in 98% human serum and incubated in 37 °C for 8 h. For systemic infection, *Candida albicans* was injected into mice via the lateral tail vein with the inoculum indicated.

### 4.19. Isolation of Kidney Cells

Kidneys from mice perfused with PBS were minced and digested by Collagenase and DNase I (Roche) at 37 °C for 30 min. Red blood cells were lysed with ACK lysis buffer (Lonza, Cambridge, MA, USA) and then processed for density gradient centrifugation using Percoll (GE Healthcare, Singapore). The cells at the interphase of 40% and 70% were isolated for further applications. For Dectin-1^+^ monocytic cell isolation, Dispase ll was also added to the digestion solution, and the cell suspension was stained with anti-Dectin-1- and Dectin-2-Alexa 647 mAb (AbD Serotec, Kidlington, UK) after FcR blocking for sorting with FACSAriall (BD Biosciences).

### 4.20. House Dust Mite (HDM) Extract-Induced Allergy Model

Mice were first intraperitoneally injected with HDM extract (10 ng Der p1/mouse; GREER laboratories) (Day-7). From day 0, mice were intranasally administered HDM extract (25 μg of whole protein/25 μL per mouse) under anesthesia daily for 8 days and sacrificed 4 h after the last administration. Equivalent amount of blood, bronchoalveolar lavage (BAL) fluid, and lung tissue were isolated for further analysis. For lung mononuclear cell isolation, a Lung Dissociation Kit (Miltenyi Biotec) and a gentleMACS Dissociator (Miltenyi Biotec) was used according to the manufacturer’s instructions. Cell suspension was further processed for lymphocyte isolation using Percoll (GE Healthcare). Cell suspension from BAL fluid was stained with CD11b-BV605, CD11c-PE, Ly6G-PerCP-Cy5.5, SiglecF-APC, TCRb-BV510, CD4-BV605, together with Fixable Viablity Dye eFluor780 (Invitrogen) and flow cytometry was performed by FACSFortessa (BD Biosciences) and analyzed with FlowJo software (BD Biosciences). Antibodies were purchased from Biolegend. The actual numbers of each cell population were calculated from total BAL cell number and percentage obtained from FACS analysis.

### 4.21. Histology

Lung from HDM extract-induced allergic mice was collected and fixed in 10% formaldehyde. Fixed lungs were then processed for PAS staining.

### 4.22. Statistical Analysis

Statistical significance was calculated using Prism 9 (GraphPad Software version 9) as indicated in the legend. *p* < 0.05 was considered significant in the present experiments (other than microarray).

## Figures and Tables

**Figure 1:**
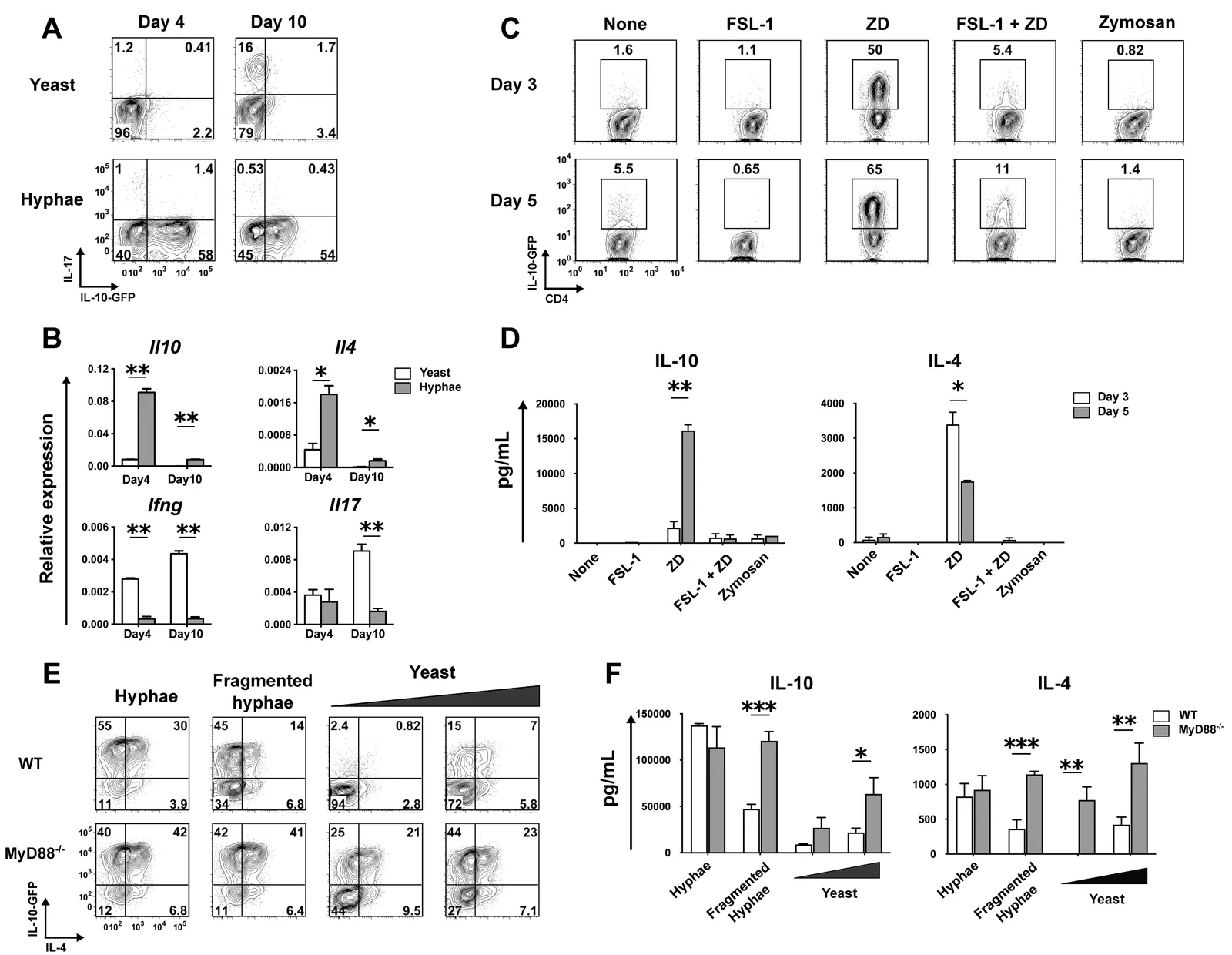
***C. albicans*****-derived Dectin-1 stimulation induces IL-10 production from CD4^+^ T cells.** (**A**,**B**) Naïve CD4^+^ T cells isolated from IL-10-GFP mice were stimulated with BMDCs by anti-CD3 mAb in the presence of indicated form of heat-killed *C. albicans* and cells were analyzed at days 4 and 10 by flow cytometry (**A**) and qPCR (**B**). (**C**,**D**) Naïve CD4^+^ T cells isolated from IL-10-GFP mice were stimulated as in (**A**) in the presence of Dectin-1 and/or TLR2 ligands and analyzed at days 3 and 5 by flow cytometry (**C**) and ELISA (**D**) of culture supernatants; ZD-stimulated CD4^+^ T cells producing high levels of IL-10 under these conditions were defined as Dect-10 T cells. (**E**,**F**) Naïve CD4^+^ T cells isolated from IL-10-GFP were stimulated by wild type or MyD88^-/-^ BMDC in the presence of *C. albicans* yeast, hyphae, or fragmented hyphae and analyzed at day 4 by flow cytometry (**E**) and ELISA (**F**) of culture supernatant. Data are representative of biological replicates (*n* = 2) from 3 independent experiments. Error bars represent SD. * *p* < 0.05, ** *p* < 0.01, *** *p* < 0.001 (unpaired two-tailed *t* test).

**Figure 2:**
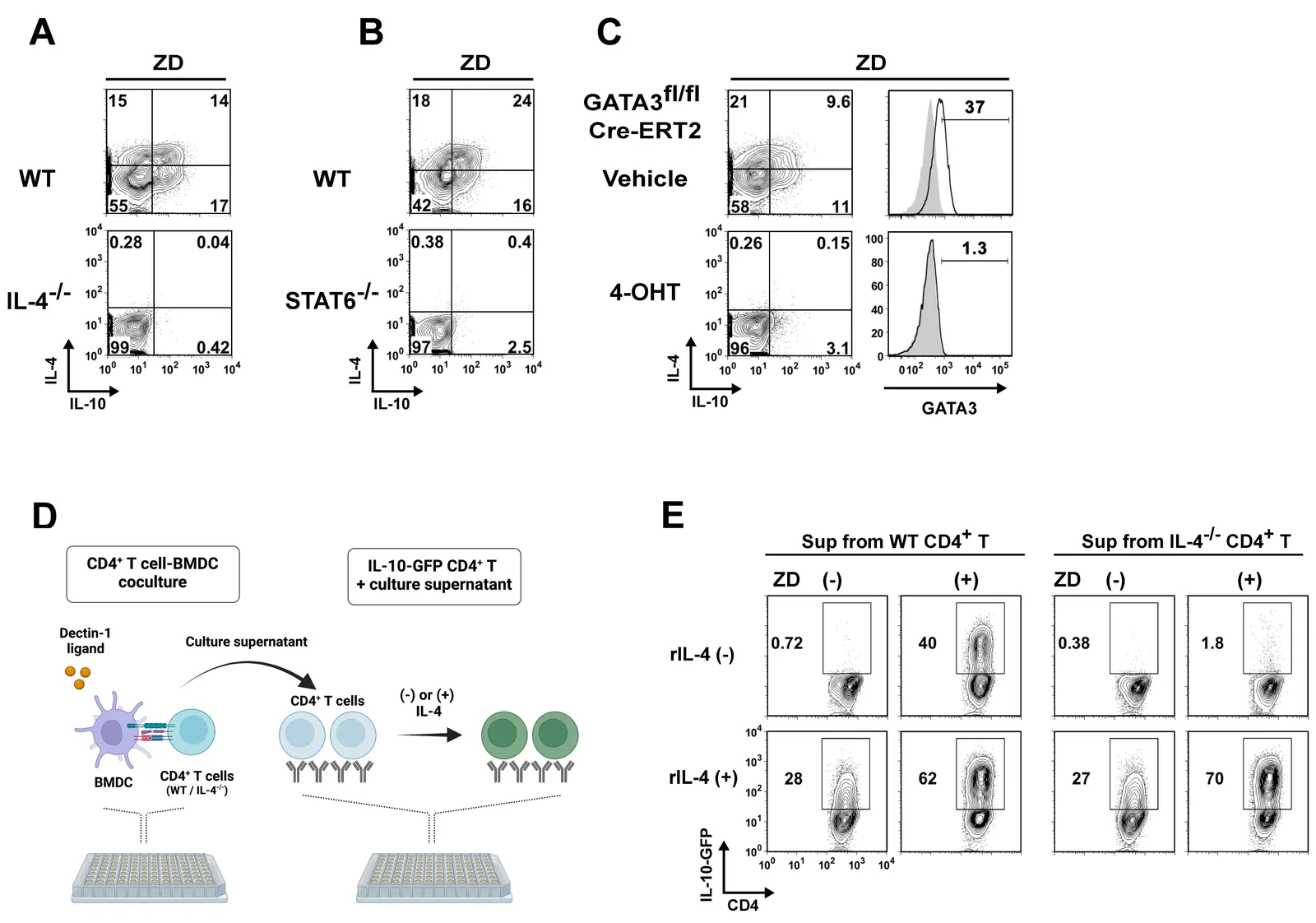
**High IL-10 production by T cells induced by dendritic cells stimulated via Dectin-1 requires initial T cell IL-4 expression.** (**A**–**C**) Naïve CD4^+^ T cells from IL-4^-/-^ (**A**), STAT6^-/-^ (**B**), GATA3^flox/flox^-Cre-ERT2 in the addition of 4-hydroxy tamoxifen (4-OHT) (**C**) were stimulated with BMDCs by anti-CD3 mAb in the presence of ZD; cells were analyzed by flow cytometry at day 5. (**D**) ChIP assays were performed using anti-acetyl histone 3 antibody with cells indicated in (**C**) and processed for qPCR using primers targeting the IL-10 promoter region; fold enrichment was calculated against values of isotype control. (**D**) Graphical scheme of procedures conducted. Supernatants from WT- or IL-4^-/-^-CD4^+^ T/BMDCs co-culture in the presence or absence of ZD were added to IL-10-GFP naïve CD4^+^ T cell culture stimulated with plate-bound CD3 mAb and soluble CD28 mAb with or without the addition of recombinant IL-4 (10 ng/mL). Scheme Created in BioRender. Tajima, M. (2026) https://BioRender.com/cctf1nd. (**E**) CD4^+^ T cells stimulated in the presence of culture supernatant as indicated in (**D**) were analyzed by flow cytometry at day 4. Data are representative of biological replicates (*n* = 2) from 3 independent experiments. Error bars represent SD (unpaired two-tailed *t* test).

**Figure 3:**
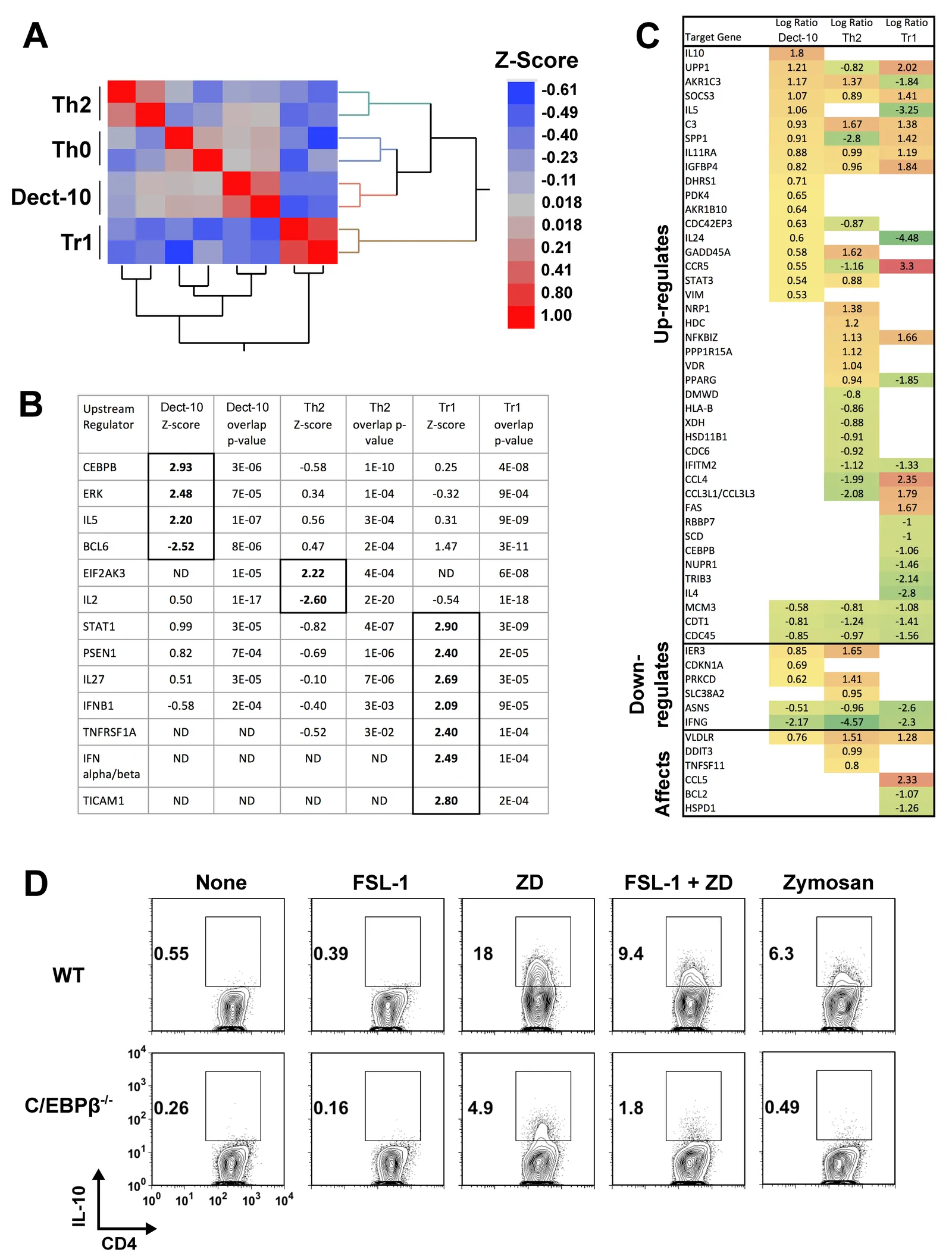
**Gene profiling established that Dect-10 T cells are a transcriptionally unique T cell subset that exhibit C/EBPβ activation.** (**A**) Correlation of whole-genome mRNA expression profiles of Th0, Th2, Tr1 and Dect-10 T cells based on principal coordinate analysis (Pearson scale). (**B**) Cell type specific upstream regulators selected using overlap *p*-value and activity Z-score statistical criteria that were satisfied for only one of the three cell types (shown in bold). (**C**) Microarray expression evidence for C/EBPβ as an upstream regulator. The table shows the log2 ratios for genes differentially expressed in one or more cell types, when compared to Th0 cells, that are known to be influenced by C/EBPβ activity according to the IPA database. (**D**) Naïve CD4^+^ T cells from WT or C/EBPβ^-/-^ were stimulated with WT BMDCs by anti-CD3 mAb in the presence of indicated Dectin-1 and/or TLR2 ligands and analyzed at day 5 by flow cytometry. Data are representative of biological replicates (*n* = 2) from 3 independent experiments.

**Figure 4:**
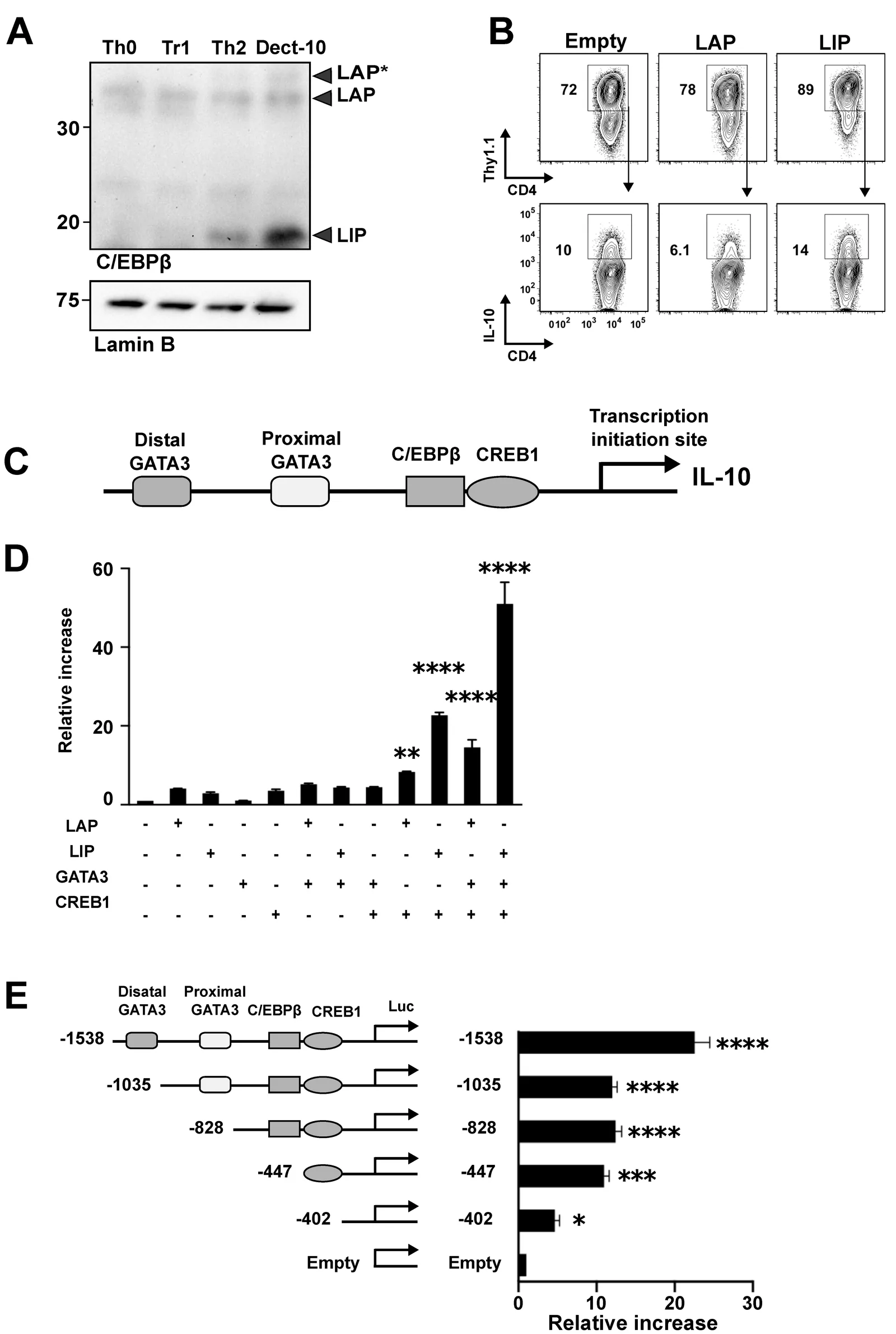
**C/EBPβ exerts a positive effect on Dect-10 T cell IL-10 production via its LIP isoform.** (**A**) Nuclear extracts of Th0, Tr1, Th2, and Dect-10 T cells were subjected for Western blotting for detection of C/EBPβ isoforms. (**B**) C/EBPβ^-/-^ naïve CD4^+^ T cells were retrovirally transduced with either C/EBPβ-LAP or -LIP marked with Thy1.1 and analyzed for IL-10 production by flow cytometry at day 5. (**C**) Diagram of C/EBPβ, CREB1, and GATA3 binding sites within the IL-10 promoter. (**D**) IL-10 promoter activity analyzed with a luciferase reporter construct expressed in HEK293T cells together with expression plasmids as indicated; dbcAMP was added 4 h after the transfection; luciferase activity was measured at 24 h after transfection and normalized against control firefly luciferase activity. Relative increase and statistical analysis were calculated against condition without expression plasmids. (**E**) Activity of truncated reporter constructs after transfection into HEK293T cells together with LIP, CREB1 and GATA3 expression plasmids treated with dbcAMP as in (**D**). Relative increase and statistical analysis calculated against empty luciferase reporter construct. Data are representative of biological replicates (*n* = 2) from 3 independent experiments. Error bars represent SD. * *p* < 0.05, ** *p* < 0.01, *** *p* < 0.001, **** *p* < 0.0001 (one-way ANOVA).

**Figure 5:**
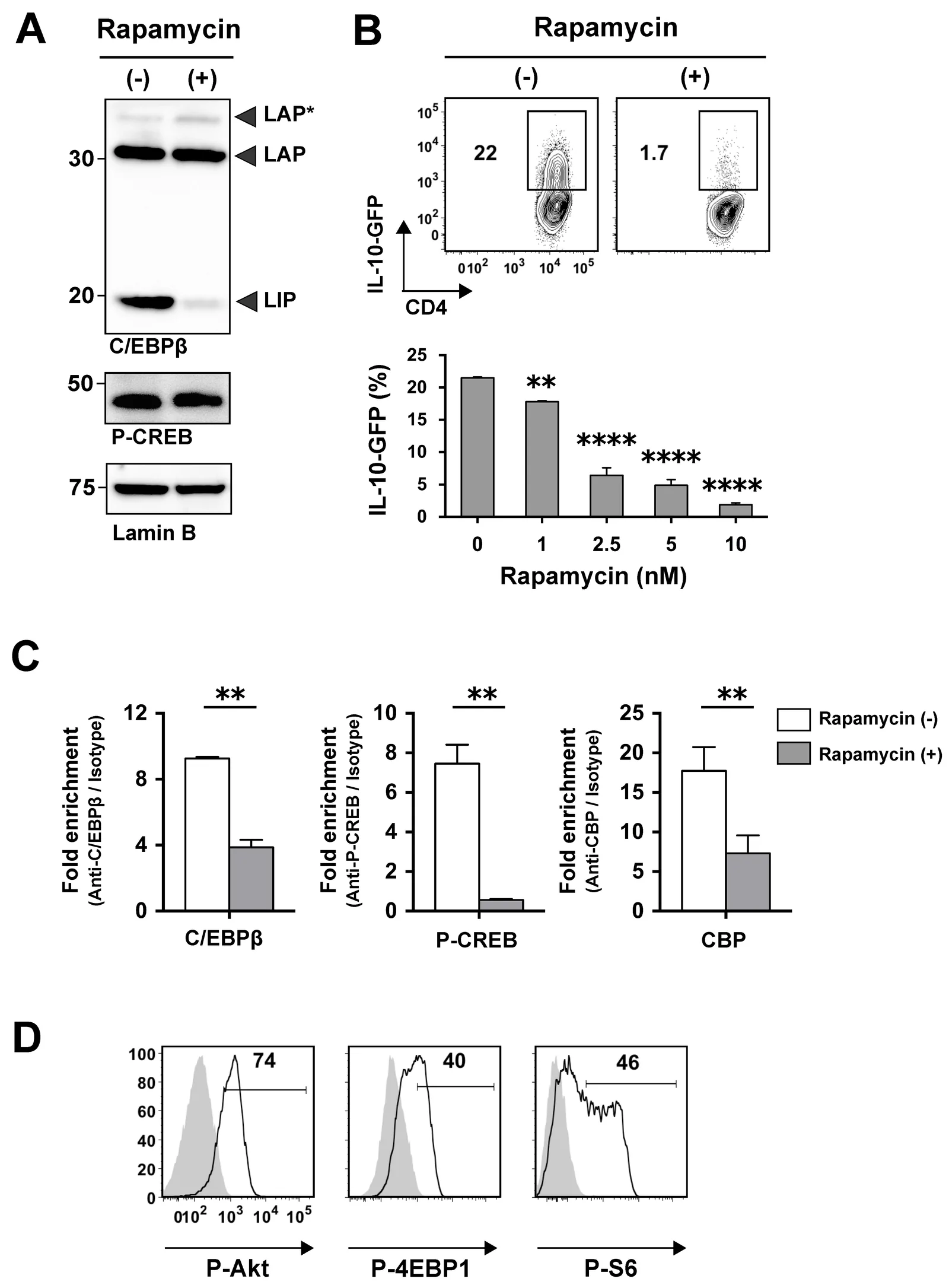
**Regulation of C/EBPβ isoform expression in Dect-10 T cells by mTOR.** (**A**) Naïve CD4^+^ T cells isolated from IL-10-GFP mice were stimulated with plate-bound CD3 mAb and soluble CD28 mAb cultured in the presence of supernatants collected from heat-killed hyphal *C. albicans*-stimulated T-BMDC cell cocultures, with or without rapamycin (10 nM) for 4 days; nuclear extracts of each cell were subjected to Western blotting for detection of C/EBPβ, phospho-CREB, and Lamin B. (**B**) Cells from (**A**) were analyzed by flow cytometry and percentage of IL-10-GFP-positive cells were calculated. Statistical analysis calculated against no rapamycin condition. (**C**) Cells from (**A**) were subjected to ChIP analysis using anti-C/EBPβ, phospho-CREB, and CBP antibodies and processed for qPCR using primers targeting each corresponding binding site within the IL-10 promoter region. (**D**) Phospho-Akt and phospho-4EBP1 level of cells stimulated as in (**A**) were analyzed by flow cytometry. Filled histogram represent isotype control staining. Data are representative of biological replicates (*n* = 2) from 3 independent experiments. Error bars represent SD. * *p* < 0.05, ** *p* < 0.01, **** *p* < 0.0001 (one-way ANOVA for (**B**) and unpaired two-tailed *t* test for (**C**)).

**Figure 6:**
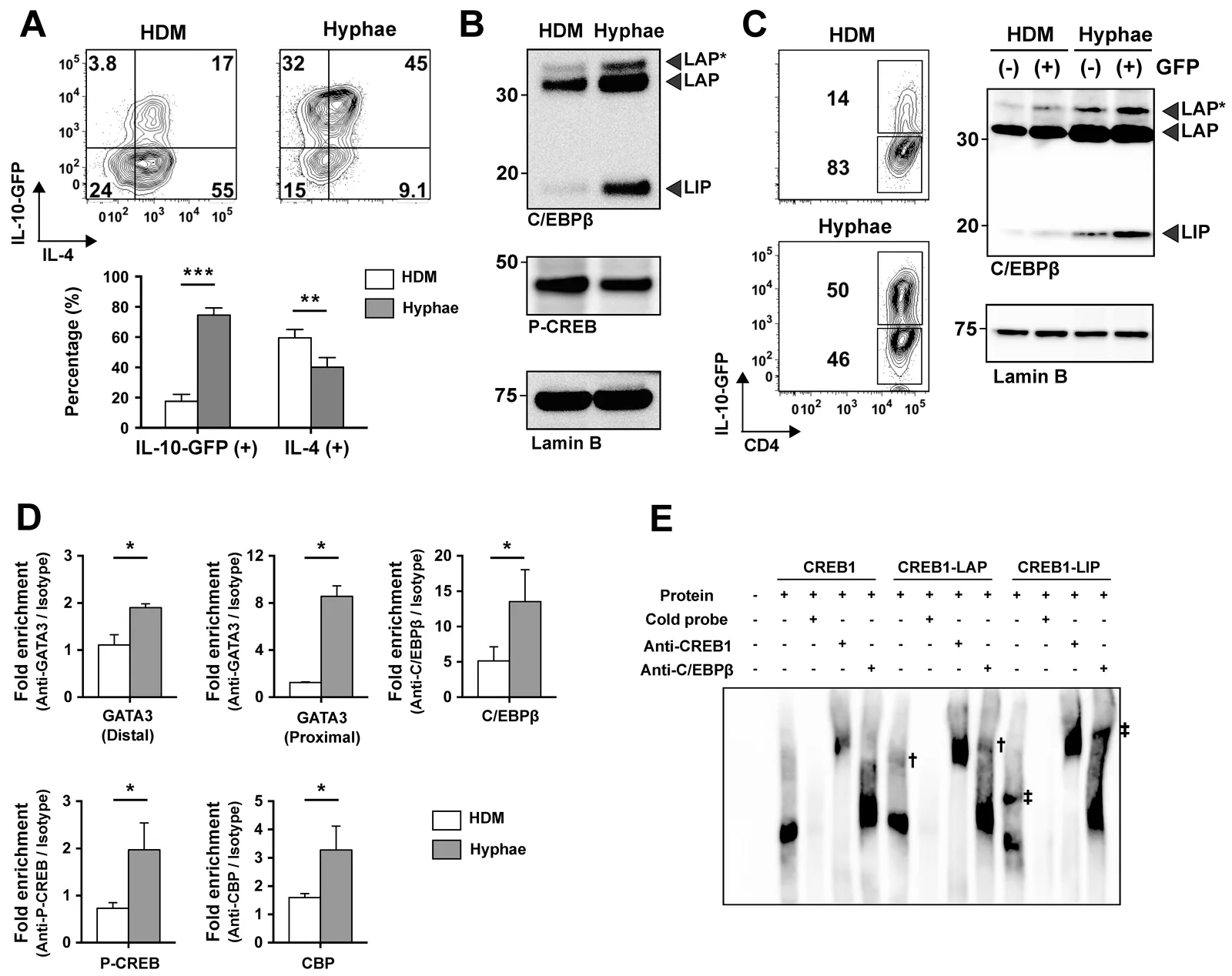
**The C/EBPβ isoform LIP is associated with IL-10 transcription in Dect-10 T cells, but not Th2 cells, and enhances CREB1 binding to its binding site.** (**A**) Naïve CD4^+^ T cells isolated from IL-10-GFP mice were stimulated with plate-bound CD3 mAb and soluble CD28 mAb in the presence of HDM extract or heat-killed hyphal *C. albicans* and analyzed at day 4. Percentages of IL-10-GFP- and IL-4-positive cells were calculated. (**B**) Nuclear fraction of cells stimulated as in (**A**) was subjected to Western blotting. (**C**) Nuclear extracts of IL-10-GFP-positive (+) and -negative (-) cells from CD4+ T cells stimulated as in (**A**) were subjected to Western blotting. (**D**) Dect-10 T cells were subjected to ChIP assays and processed for qPCR. (**E**) EMSA studies of protein obtained from HEK293T cells transfected for FLAG-CREB1 alone, or with either FLAG-LAP or FLAG-LIP, and then immunoprecipitated. The amount of protein was adjusted to equalize the CREB1 level. Samples were then incubated with an oligonucleotide probe flanking the CREB1-C/EBPβ binding site in the IL-10 promoter. Signals for CREB/LAP complex binding to the oligonucleotide are marked with † and the CREB/LIP complex with ‡. Data are representative of biological replicates (*n* = 2) from 3 independent experiments. Error bars represent SD. * *p* < 0.05, ** *p* < 0.01, *** *p* < 0.001 (unpaired two-tailed *t* test).

**Figure 7:**
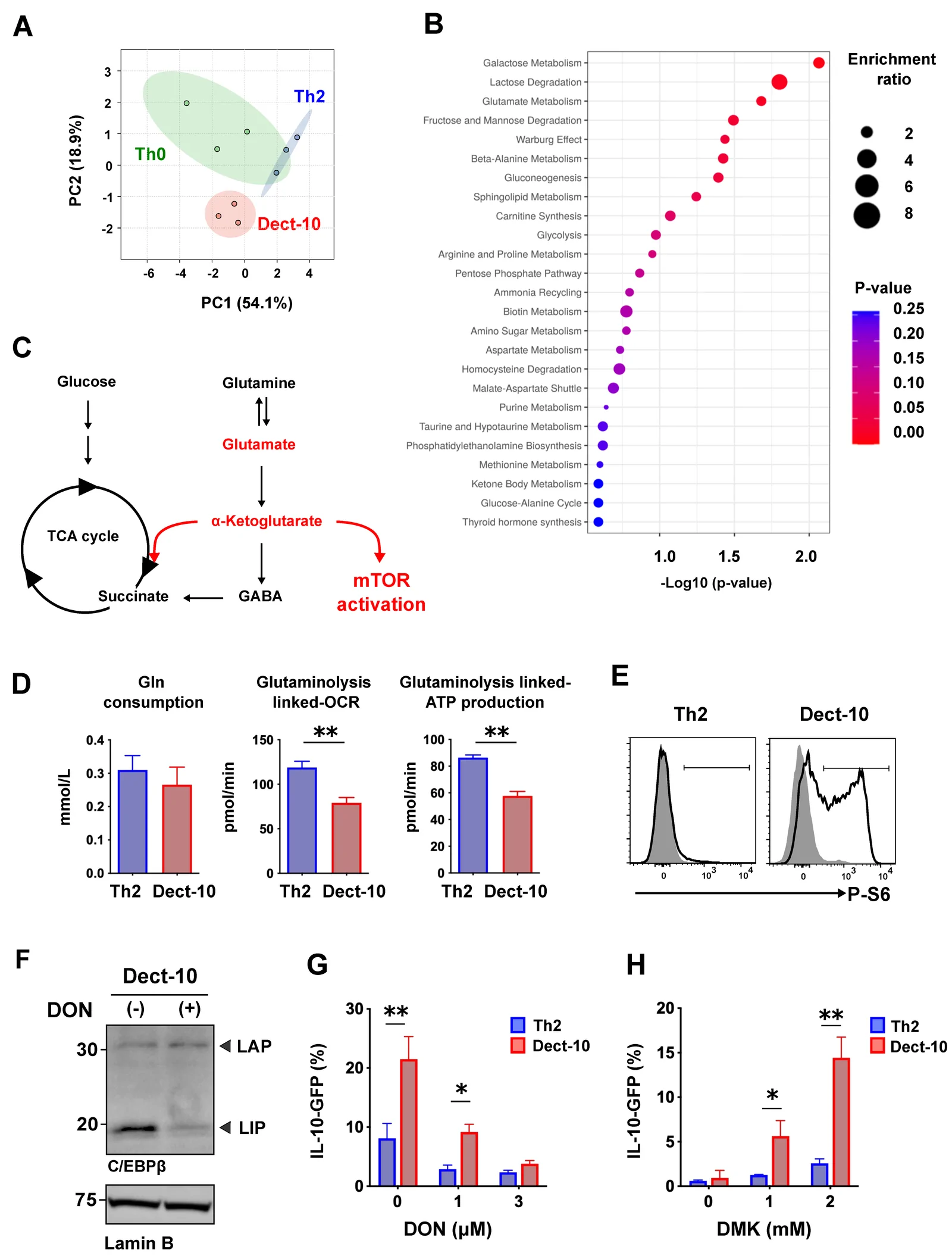
**mTOR activity by glutaminolysis-induced α-KG in Dect-10 T cells.** (**A**) IL-10-GFP naïve CD4^+^ T cells were stimulated with plate-bound CD3 mAb and soluble CD28 mAb in the presence of 1 ng/mL IL-4 (Th2), heat-killed hyphae-treated T-BMDC coculture supernatants (Dect-10 T cells) or without further treatment (Th0), and PCA was conducted from metabolome analysis at day 4. Symbols represent the results of biological replicates (*n* = 3). (**B**) Metabolite-set enrichment analysis was performed from substances found in (**A**). (**C**) Scheme of glutamine metabolism focused on the present study. (**D**) Glutamine consumption of Th2/Dect-10 T cells stimulated as in (**A**) measured after 48 h (*n* = 3). Glutaminolysis-linked OCR/ATP were measured by flux analyzer and calculated by subtracting the OCR/ATP level of cells cultured in Gln-free media from the Gln(+) cells (*n* = 6). (**E**) Phospho-S6 level of Th2/Dect-10 T cells induced as in (**A**) were analyzed by FACS analysis. Filled histogram represent isotype control staining. (**F**) C/EBPβ expression pattern of Dect-10 T cells induced as in (**A**) treated with DON (3 μM) was analyzed by Western blotting. (**G**) IL-10-GFP-positive cells from Th2/Dect-10 T cells induced as in (**A**) treated with DON in indicated concentration were analyzed by FACS. (**H**) IL-10-GFP-positive cells from Th2/Dect-10 T cells induced as in (**A**) in glutamine-deprived condition in the presence of indicated concentration of DMK. Representative data are shown from 3 independent experiments (*n* represents biological replicates). Error bars represent SD. * *p* < 0.05, ** *p* < 0.01 (unpaired two-tailed *t* test).

**Figure 8:**
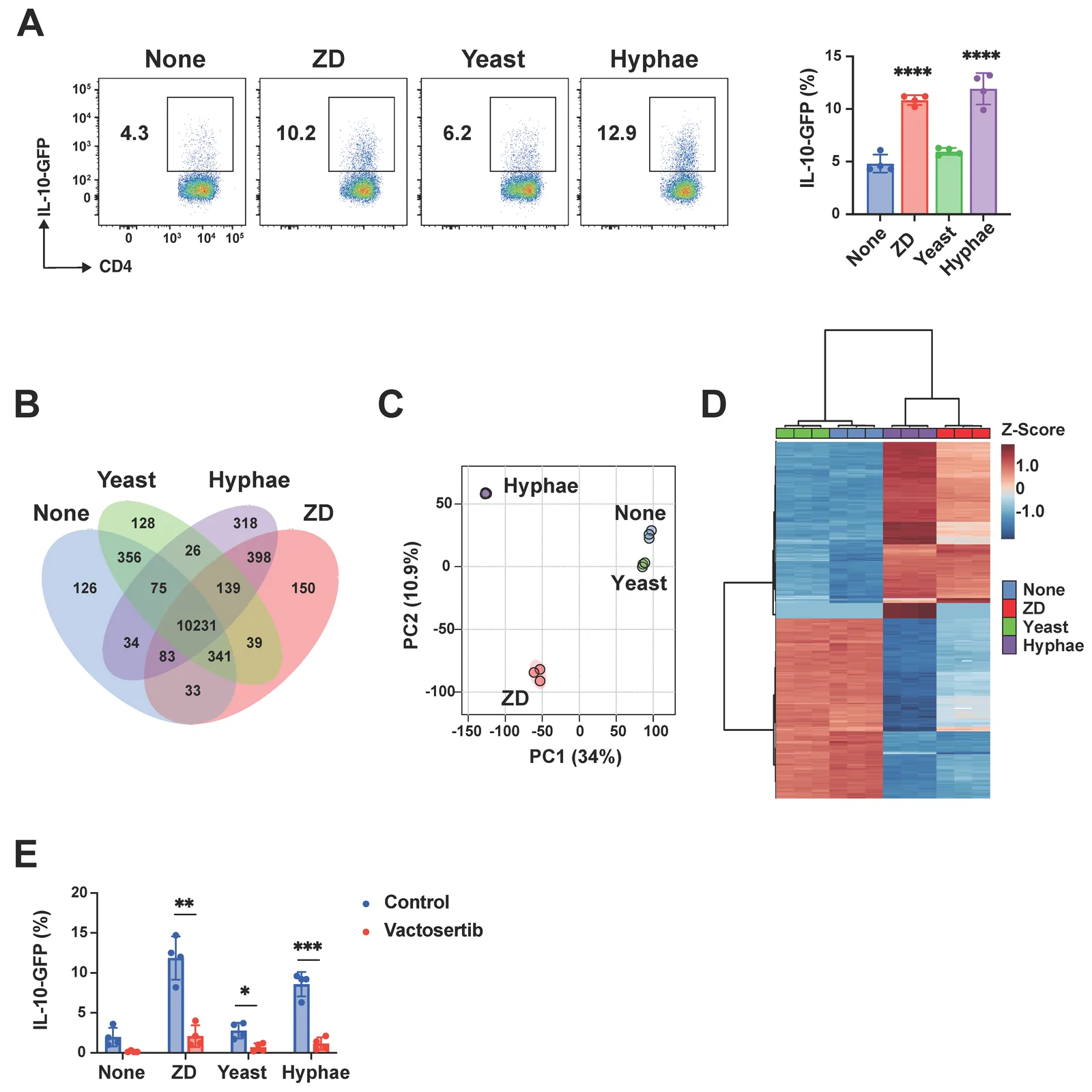
**An ActRIB (ALK4) TGF-β receptor family ligand produced by Dectin-1-stimulated DCs induce Dect-10 T cell.** (**A**) Supernatants of BMDCs cultured in the presence of Zymosan Depleted (ZD), yeast form, hyphal form of heat-killed *C. albicans*, or control media were collected at day 3. Collected supernatant was added to culture of IL-10-GFP naive CD4^+^ T cells stimulated with plate-bound CD3 mAb and soluble CD28 mAb for 4 days and analyzed by FACS. (Left) Representative FACS plots of IL-10-GFP CD4^+^ T cells. (Right) Bar graph of IL-10-GFP-positive cells (%). Symbols represent the results of biological replicates (*n* = 4). (**B**) Venn diagram of DEG number analyzed by RNAseq across four BMDC groups stimulated with indicated components for 2 days. (**C**) PCA of first two principal components of RNAseq data. PC1 and PC2 explains 34% and 10.9% of the total variance, respectively. Symbols represent the result of biological replicates (*n* = 3). (**D**) Heatmap generated from top 300 ranked DEGs within the comparison across the 4 groups (one-way ANOVA) together with hierarchical clustering by Euclidean distance. (**E**) IL-10-GFP naive CD4^+^ T cells were stimulated as in (**A**) in the presence of vactosertib (10 μM) for 4 days and analyzed by flow cytometry. Bar graphs of IL-10-GFP-positive cells. Symbols represent the result of biological replicates from 3 independent experiments (*n* = 4). Error bars represent SD. * *p* < 0.05, ** *p* < 0.01, *** *p* < 0.001, **** *p* < 0.0001 (unpaired two-tailed *t* test).

**Figure 9:**
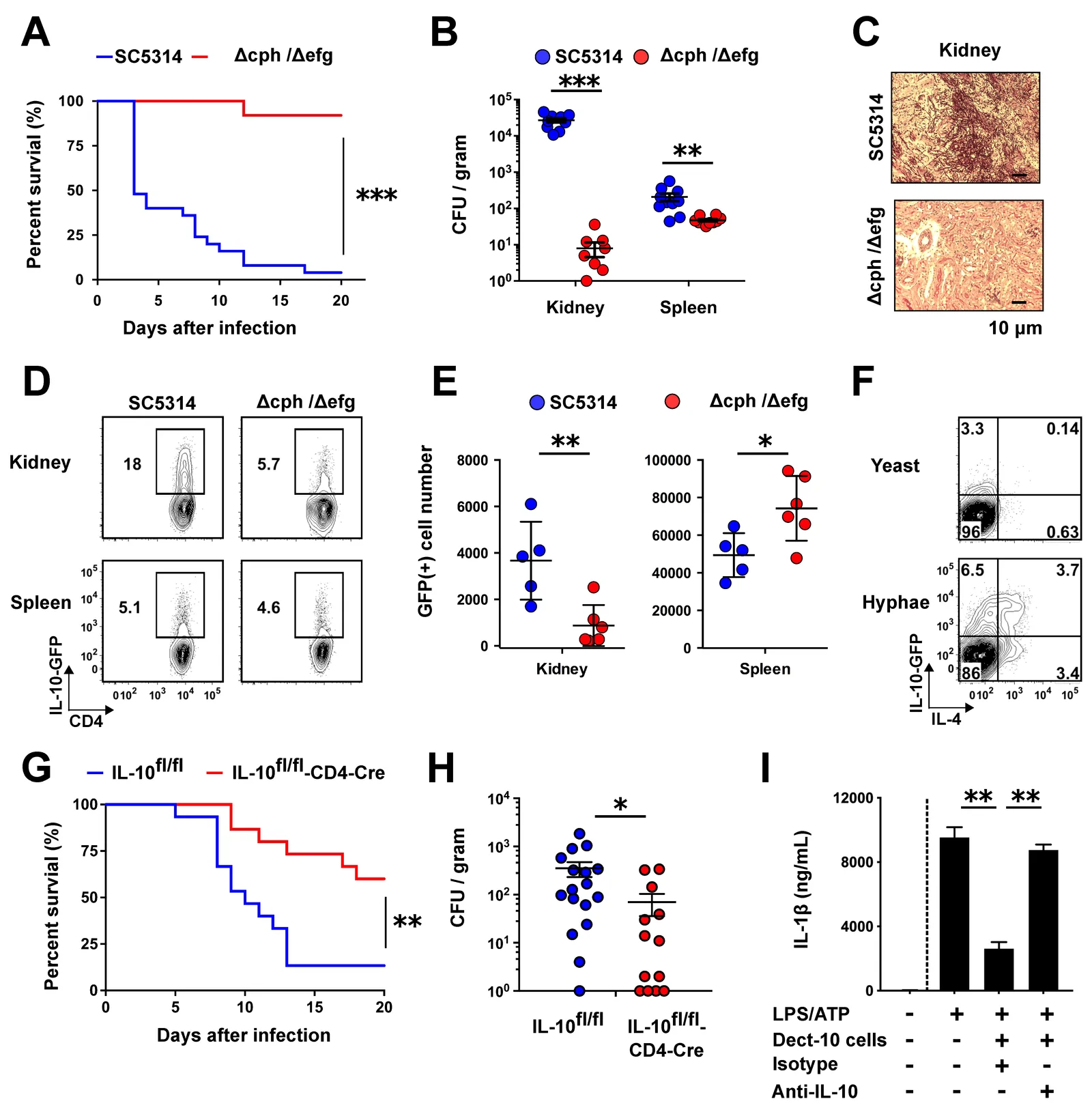
**Dect-10 T cells are induced in systemic candidiasis kidney and negatively impacts the survival of infected host.** (**A**–**C**) WT C57BL/6J mice were intravenously injected SC5314 or Δcph/Δefg (5 × 10^5^/mouse). Survival rate (*n* = 25) (**A**), fungal burden in the kidney and spleen at day 5 (*n* = 10) (**B**), and periodic acid–Schiff staining of the kidney from each group at day 5 (**C**) are shown. Symbols represent the results of biological replicates. (**D**) Kidney and spleen cells from infected IL-10-GFP mice were subjected to flow cytometry at day 5 (*n* = 5). Representative FACS plots are shown. (**E**) Quantitation of IL-10-GFP-positive CD4^+^ T cells from (**D**). Symbols represent the results of biological replicates (*n* = 5). (**F**) Dectin-1/2^+^ cells from kidney and spleen were cultured with OT-II/IL-10-GFP naïve CD4^+^ T cells and stimulated by OVA Class II peptide in the presence of heat-killed yeast or hyphal *C. albicans*. Representative FACS plots are shown. (**G**) Survival rate of IL-10^flox/flox^ (*n* = 15) and IL-10^flox/flox^ CD4-Cre (*n* = 15) mice infected by SC5314 (1 × 10^5^/mouse). (**H**) Kidney fungal burden of IL-10^flox/flox^ (*n* = 17) and IL-10^flox/flox^ CD4-Cre (*n* = 13) 7 days after infection. Symbols represent the results of biological replicates. (**I**) BMDMs were cultured as indicated and IL-1β level was measured by ELISA. Data are representative of biological replicates (*n* = 2) from 3 independent experiments. Error bars represent SD. Statistical analysis calculated within LPS/ATP-treated conditions. Error bars represent SEM and data were pooled from 3 independent experiments otherwise indicated. * *p* < 0.05, ** *p* < 0.01, *** *p* < 0.001 (unpaired two-tailed *t* test for (**B**,**E**,**H**), one-way ANOVA for (**I**), and Gehan-Breslow-Wilcoxon test for (**A**,**G**)).

## Data Availability

The microarray and RNAseq data have been deposited in NCBI’s Gene Expression Omnibus and are accessible through GEO Series accession number GSE87673 and GSE316343. The raw data supporting the conclusions of this article will be made available by the authors on request.

## Supplementary Materials

The following supporting information can be downloaded at https://www.mdpi.com/article/10.3390/ijms27198447/s1.
