# Supplementary material for: Glutaminolysis and mTOR-C/EBPβ Signaling Drives the Differentiation of Novel IL-10-Producing Regulatory T Cells
Source: Int J Mol Sci. 2026 Sep 22;27(19):8447. doi: 10.3390/ijms27198447 (PMC13664842; doi:10.3390/ijms27198447)
Supplement: Supplementary file 1 [file ijms-27-08447-s001.zip › 260922 Supplemental Information.pdf]

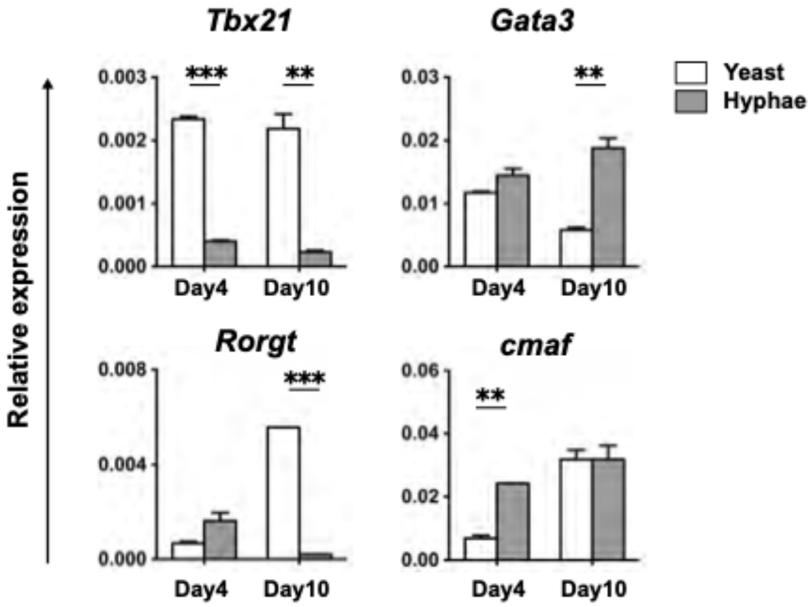

Supplementary Figure S1. Expression of transcription factor in yeast- or hyphal-*C. albicans* stimulated CD4$^+$ T cells.

Naïve CD4$^+$ T cells were stimulated with BMDCs by anti-CD3 mAb (0.2 μg/mL) in the presence of yeast- or hyphal *C. albicans* and processed for qPCR. Data are representative of biological replicates (n=2) from 3 independent experiments. Error bars represent SD. *p<0.05, **p<0.01, ***p<0.001. (unpaired two tailed t test).

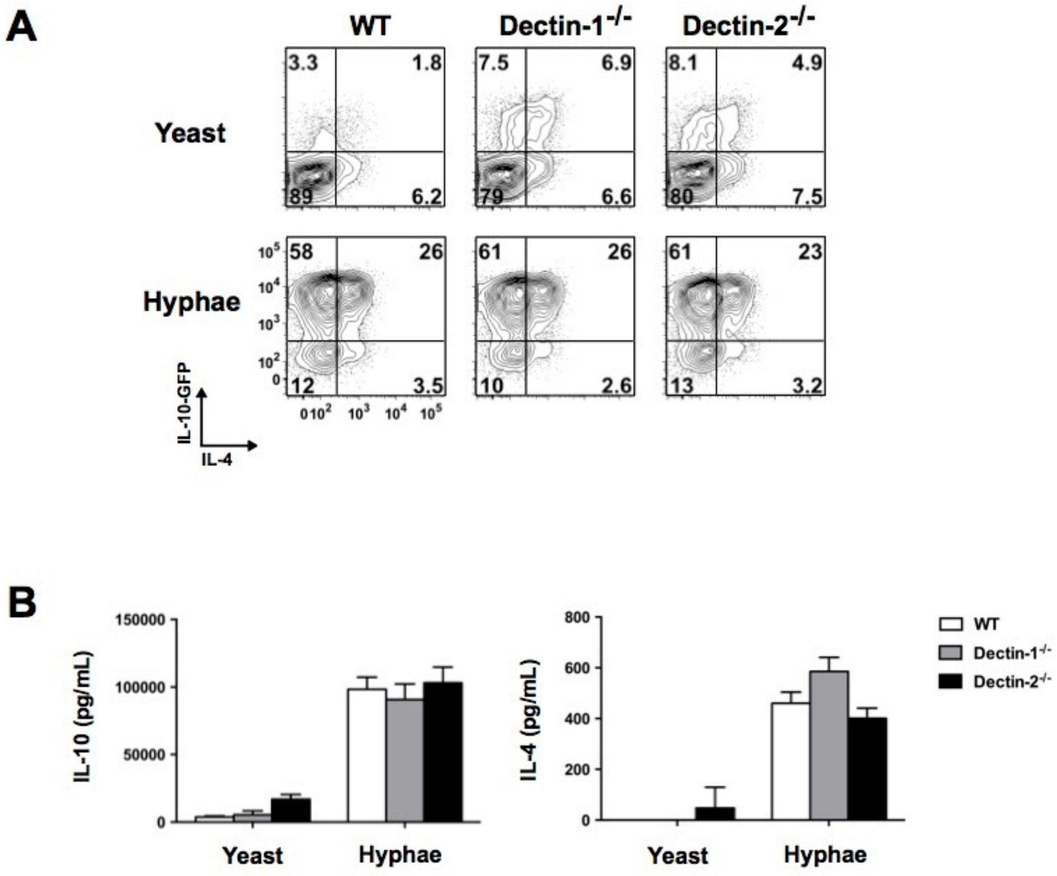

Supplementary Figure S2. Dectin-1 and Dectin-2 have redundant role in *C. albicans* recognition and induction of Dect-10 cells.

(A, B) Naïve $CD4^+$ T cells isolated from IL-10-GFP mice were stimulated with wild type, Dectin-$1^{-/-}$, or Dectin-$2^{-/-}$ BMDCs by anti-CD3 mAb (0.2 μg/mL) in the presence of yeast- or hyphal *C. albicans* and analyzed at day 5 by flow cytometry (A) and ELISA (B) of culture supernatant. Data are representative of biological replicates (n=2) from 3 independent experiments. Error bars represent SD.

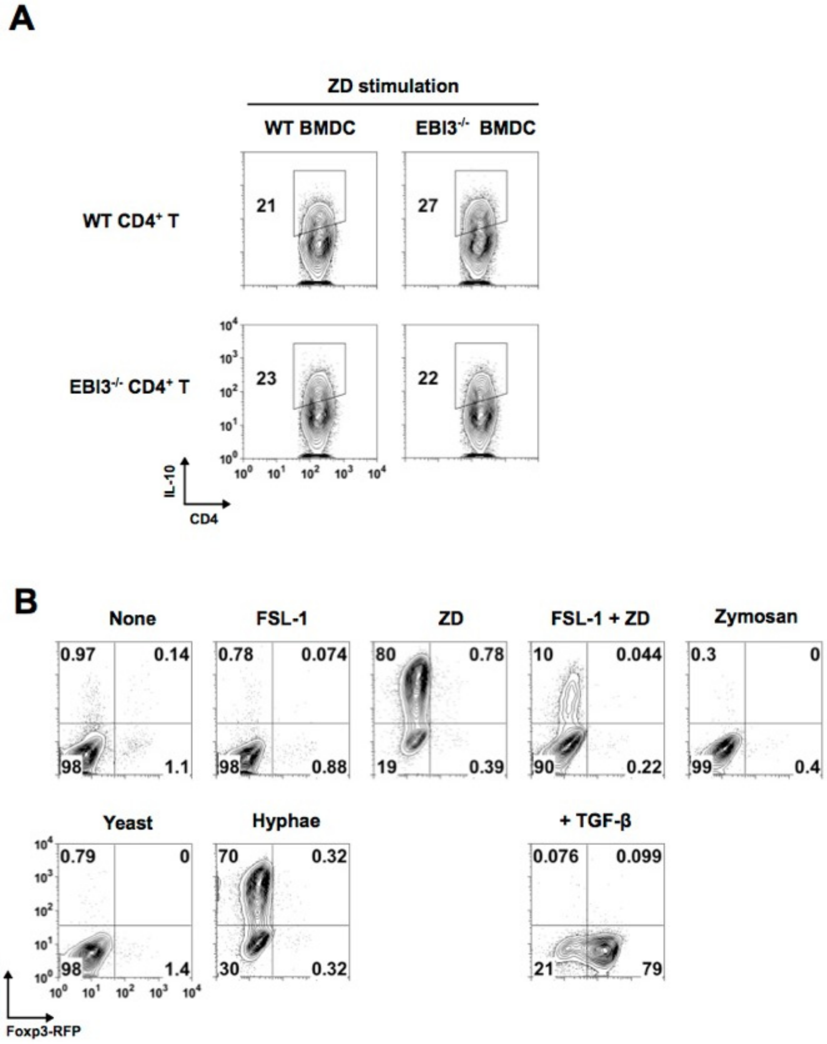

Supplementary Figure S3. Dect-10 T Cells are distinct from Tr1 and Treg cells.
(A) Wild type or EBI3$^{-/-}$ naïve CD4^{+} T cells were stimulated by either wild type or EBI3$^{-/-}$ BMDCs by anti-CD3 mAb (0.2 μg/mL) in the presence of ZD and then analyzed at day 5 by flow cytometry. (B) Naïve CD4^{+} T cells isolated from IL-10-GFP/Foxp3-RFP mice were stimulated with wild type BMDC by anti-CD3 mAb (0.2 μg/mL) in the presence of indicated ligands, or yeast- or hyphal *C. albicans*. Cells cultured with TGF-β (5 ng/mL) served for generation of Foxp3-positive cells. Data are representative of biological replicates (n=2) from 3 independent experiments.

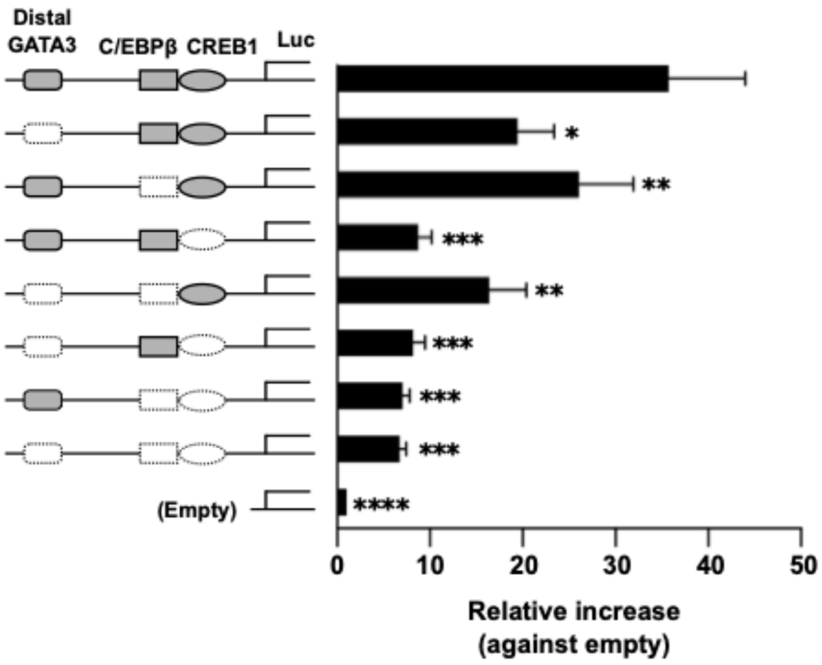

Supplementary Figure S4. Distal GATA3, C/EBPβ, and CREB1 binding to the IL-10 promoter region is critical for the maximum transcription of *Il10*.

Promoter activity analyzed by HEK293T cells transfected with an IL-10 promoter-luciferase reporter constructs that lacks various combinations of GATA3, C/EBPβ, and CREB1 binding sites together with plasmids expressing GATA3, C/EBPβ-LIP, and CREB1; dbcAMP was added 4 hrs after the transfection; Luciferase activity was measured at 24 hrs after transfection, and normalized against the control firefly luciferase activity. Relative increase was calculated against IL-10-promoter empty reporter construct. Data are representative of biological replicates (n=2) from 3 independent experiments. Error bars represent SD (n=2). *$p<0.05$, **$p<0.01$, ***$p<0.001$, ****$p<0.0001$ (one-way ANOVA against intact IL-10 promoter). Filled shapes represent intact and dotted shapes represent deleted region.

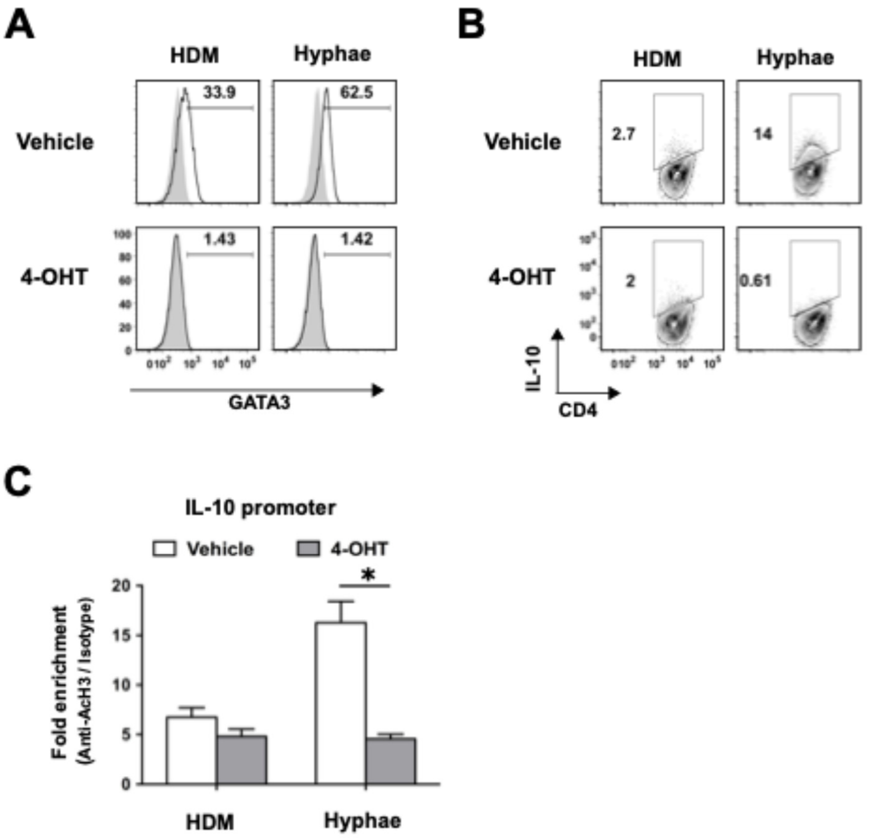

Supplementary Figure S5. GATA3 regulates the chromatin configuration of IL-10 promoter in Dect-10 cells.

(A, B) GATA3$^{fl/fl}$-Cre-ERT2 naïve CD4^{+} T cells were stimulated with BMDCs by anti-CD3 mAb (0.2 μg/mL) in the presence of HDM or hyphal-*C. albicans* with or without 4-hydroxy tamoxifen (4-OHT); cells were analyzed by flow cytometry on day 5. Filled histogram represent isotype control staining. (C) ChIP assay of cells indicated in (A) using anti-acetyl histone 3 antibody and qPCR using primers targeting the IL-10 promoter region. Data are representative of biological replicates (n=2) from 3 independent experiments. Error bars represent SD. *p<0.05 (unpaired two tailed t test).

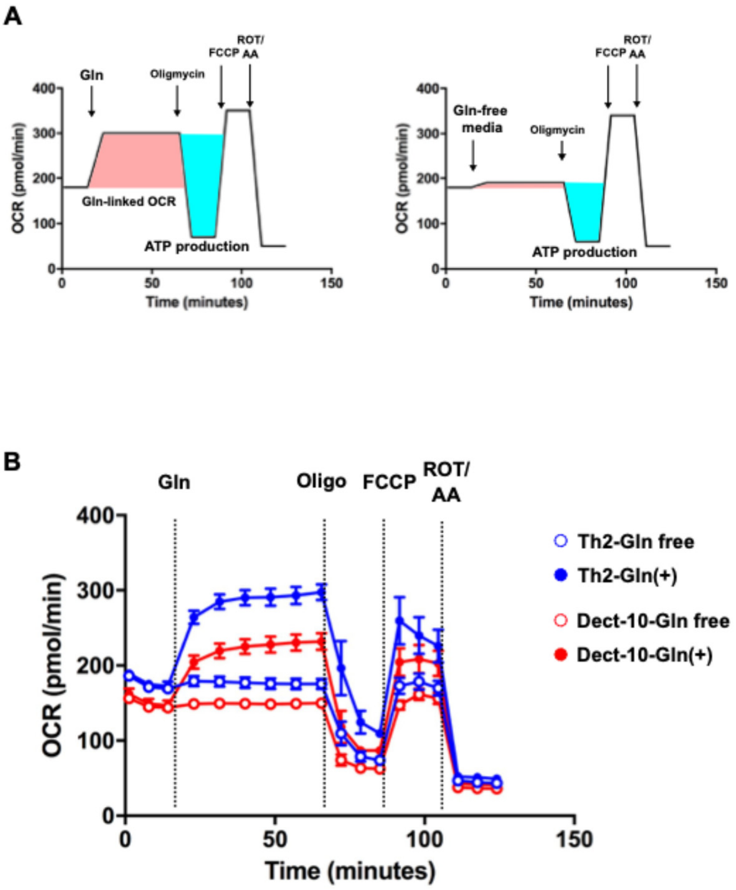

Supplementary Figure S6. Effect of glutamine metabolism on oxygen consumption rate of Th2 and Dect-10 cells

(A) Illustration of glutamine effect on oxygen consumption in Th2 and Dect-10 cells. OCR was measured in glutamine sufficient (left) or glutamine free (right) conditions. Glutaminolysis-linked OCR of Th2 and Dect-10 cells were calculated by subtracting the OCR level of cells cultured in Gln-free media from the Gln (+) cells using the measurement before Oligomycin addition. ATP production rate of Th2 and Dect-10 cells were calculated by subtracting the value of Gln free- or Gln(+)-OCR measurement as follow ((Measurement before Oligmoycin injection)-(Measurement before FCCP injection)). (B) Measurement used for glutaminolysis linked-OCR and -ATP production in Figure 7D. Data are representative of biological replicates (*n*=6) from 3 independent experiments.

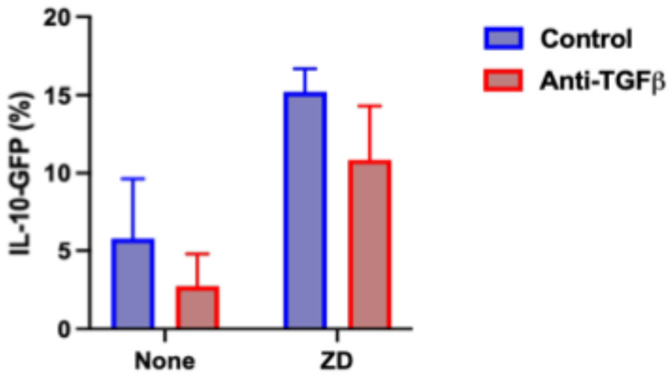

Supplementary Figure S7. Effect of anti-TGFβ neutralizing antibody on Dect-10 T cell induction

Naïve $CD4^+$ T cells isolated from IL-10-GFP mice were stimulated with plate-bound CD3 mAb and soluble CD28 mAb cultured in the presence of supernatants collected from ZD stimulated T-BMDC cell cocultures, with or without anti-TGFβ neutralizing antibody (2.5 μg/mL) for 4 days and analyzed by flow cytometry. Data are representative of biological replicates (*n*=4) from 2 independent experiments. Error bars represent SD (unpaired two tailed t test).

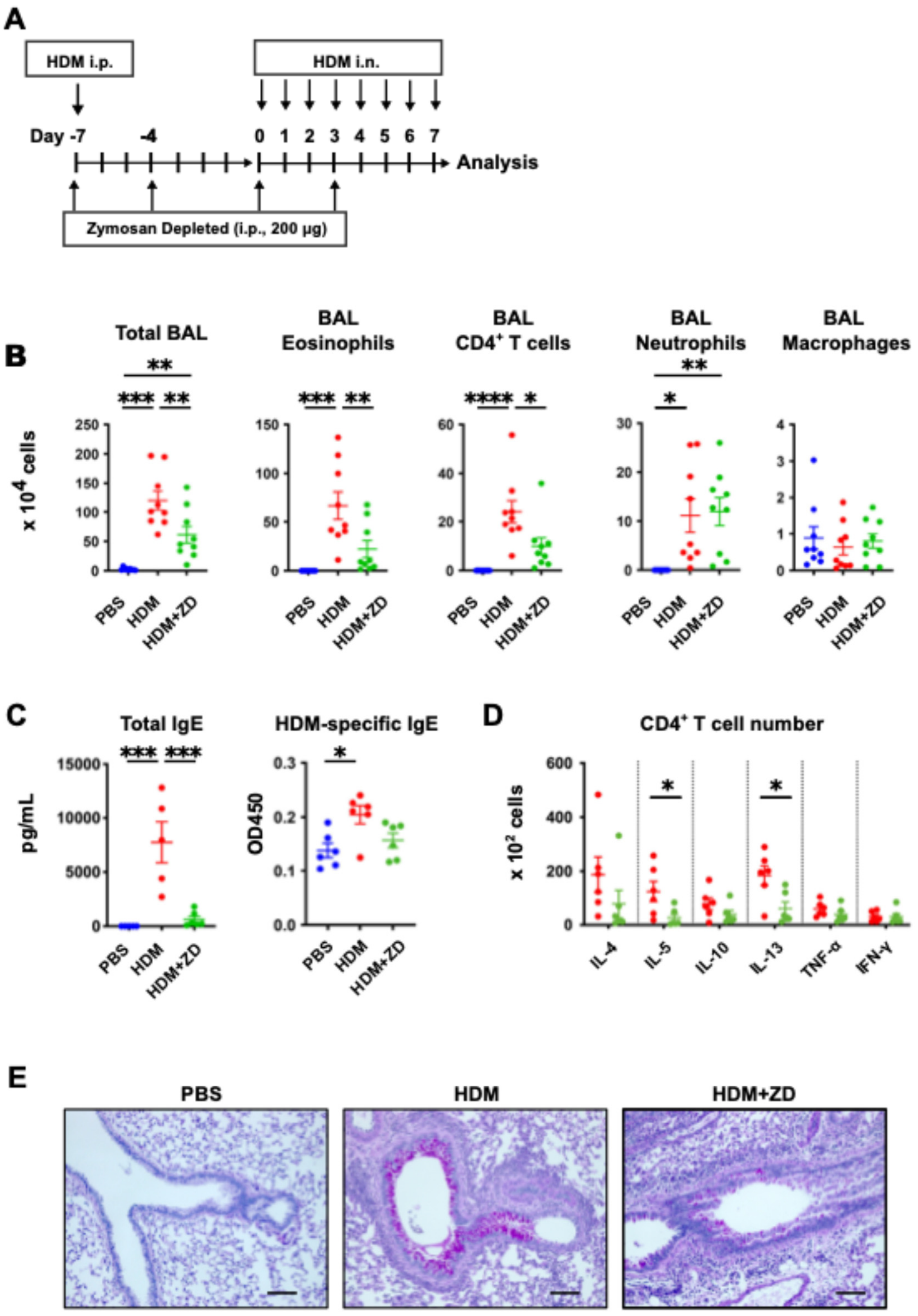

Supplementary Figure S8. Dectin-1 stimulation ameliorates house dust mite-induced allergy

(A) HDM induced allergy was induced as indicated. Mice were euthanized 4 hours after the final HDM administration (*n*=9 for each group). (B) Bronchoalveolar lavage fluid from (A) was collected and total BAL cell number was calculated. Number of BAL eosinophils, $CD4^+$ T cells, macrophages, and neutrophils were calculated from flow cytometry analysis. Symbols represent the results of biological replicates (*n*=9) (C) Plasma was collected on the day of analysis and processed for quantification of total IgE and HDM-specific IgE level by ELISA. Symbols represent the results of biological replicates (*n*=5-6). (D) Cells isolated from BAL fluid on day 7 were stimulated for intracellular cytokine staining. Percentage of IL-4/IL-5/IL-10/IL-13/TNF-α/IFN-γ positive $CD4^+$ T cells are shown. Symbols represent the results of biological replicates (*n*=6). (E) Lung was collected and processed for PAS staining. Representative image is shown for each condition. Scale bar represent 100 μm. Representative data are shown from two independent experiments. Error bars represent SEM. *p<0.05, **p<0.01, ***p<0.001, ****p<0.0001 (one-way ANOVA for (B) and (C), unpaired two tailed t test for (D)).

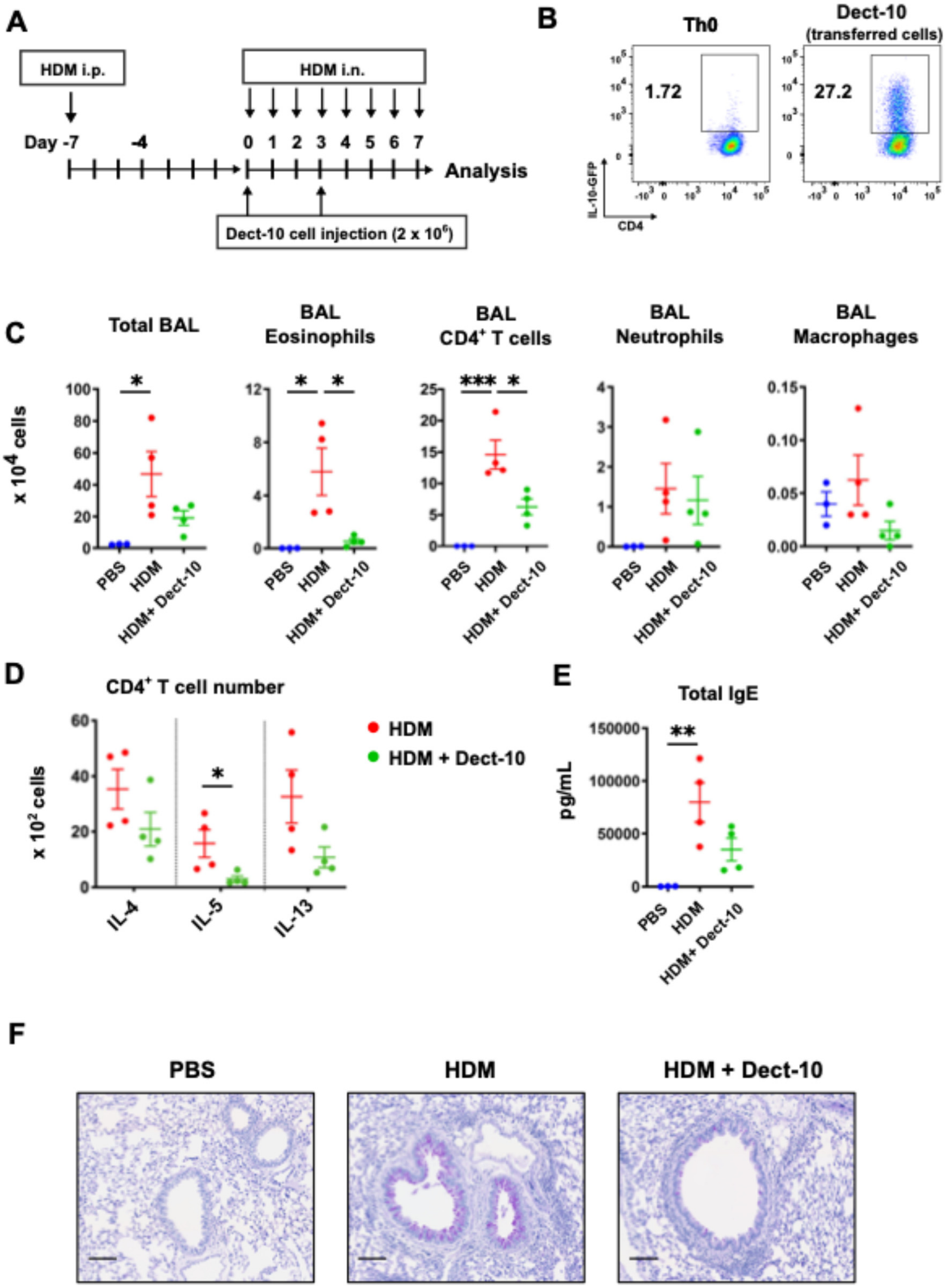
A
HDM i.p.
HDM i.n.
Day -7
-4
0 1 2 3 4 5 6 7
Analysis
Dect-10 cell injection (2 x 10^6)
B
Th0
Dect-10
(transferred cells)
1.72
27.2
IL-10-GFP
CD4
C
Total BAL
BAL Eosinophils
BAL CD4+ T cells
BAL Neutrophils
BAL Macrophages
x 10^4 cells
PBS
HDM
HDM+ Dect-10
D
CD4+ T cell number
x 10^2 cells
IL-4
IL-5
IL-13
HDM
HDM + Dect-10
E
Total IgE
pg/mL
F
PBS
HDM
HDM + Dect-10

Supplementary Figure S9. Dect-10 cells ameliorate house dust mite-induced allergy
(A) HDM induced allergy was induced as indicated. *In vitro*-induced Dect-10 cells ($2x10^6$ cells) were injected on day 0 and 3 intravenously. Mice was euthanized 4 hours after the final HDM administration. (n=3-4/group). (B) Representative FACS analysis of Dect-10 cells injected as in (A). (C) Bronchoalveolar lavage fluid was collected and total BAL cell number was calculated. Number of eosinophils, $CD4^+$ T cells, macrophages, and neutrophils were calculated from flow cytometry analysis. Symbols represent the results of biological replicates (*n*=3-4). (D) Cells isolated from BAL fluid on day 3 were stimulated for intracellular cytokine staining. Actual number of IL-4/IL-5/IL-13 positive $CD4^+$ T cells are shown. Symbols represent the results of biological replicates (*n*=4). (E) Plasma was collected on the day of analysis and processed for quantification of total IgE. Symbols represent the results of biological replicates (*n*=3-4). (F) Lung was collected and processed for PAS staining. Representative image is shown for each condition. Scale bars represent 100 µm. Representative data are shown from two independent experiments. Error bars represent SEM. *$p<0.05$, **$p<0.01$, ***$p<0.001$ (unpaired two tailed t test for (D), one-way ANOVA for (C) and (E)).

Table S1: Evidence from downstream target genes supporting predictions of cell-specific upstream regulator activity (Z-scores).

The table shows the log2 ratios for differentially expressed genes from each cell type (vs. Th0 cells) that are theoretically downstream targets of the upstream regulators shown in Table 3b. For target genes that are annotated in the IPA database as being up-regulated by CEBPB, positive expression ratios are consistent with CEBPB being more active, while negative expression ratios suggest the regulator is inhibited. Targets annotated as being down-regulated suggest the opposite inference pattern. Missing expression ratios indicate targets that did not appear in the query set for the corresponding cell type comparison; those targets were not differentially expressed and were not included in the IPA upstream regulator prediction calculation.

Table S2: Upstream regulators predicted by IPA to be responsible for the expression differences in each of the 3 cell types relative to Th0 cells.

The Z-scores test the consistency of predictions for activation ($Z > 2$) or inhibition ($Z < -2$) of the activity of the regulator as inferred from differential expression of target genes and the relationship between regulator and target according to the IPA database. The overlap p-value measures the statistical enrichment of differentially expressed genes appearing in the regulator's target gene set ("Target Molecules in the Dataset"), regardless of the annotated directionality. Here the results have been filtered for $|Z| > 2$ and p-value < 1E-3 for any of the 3 cell types. "ND" (not detected) appears for regulators for which the set of differentially expressed target genes for that cell type are less than 4.

Table S3: List of DEGs upregulated in ZD and Hyphae stimulated BMDCs.

Top 33 DEGs (t-test $P<0.05$, $|\log2FC|>0.26$ of fpkm) commonly upregulated in ZD (compared to None) and Hyphae (compared to Yeast) are listed together with the gene description.

Table S4: Primer sequences used for qPCR.
